# Endocytic trafficking factor VPS45 is essential for spatial regulation of lens fiber differentiation in zebrafish

**DOI:** 10.1242/dev.170282

**Published:** 2018-10-15

**Authors:** Toshiaki Mochizuki, Yutaka Kojima, Yuko Nishiwaki, Tetsuya Harakuni, Ichiro Masai

**Affiliations:** Developmental Neurobiology Unit, Okinawa Institute of Science and Technology Graduate University, Tancha 1919-1, Onna, Okinawa 098-0945, Japan

**Keywords:** Lens fiber differentiation, Zebrafish, VPS45, Endocytic trafficking

## Abstract

In vertebrate lens, lens epithelial cells cover the anterior half of the lens fiber core. Lens epithelial cells proliferate, move posteriorly and start to differentiate into lens fiber cells at the lens equator. Although FGF signaling promotes this equatorial commencement of lens fiber differentiation, the underlying mechanism is not fully understood. Here, we show that lens epithelial cells abnormally enter lens fiber differentiation without passing through the equator in zebrafish *vps45* mutants. VPS45 belongs to the Sec1/Munc18-like protein family and promotes endosome trafficking, which differentially modulates signal transduction. Ectopic lens fiber differentiation in *vps45* mutants does not depend on FGF, but is mediated through activation of TGFβ signaling and inhibition of canonical Wnt signaling. Thus, VPS45 normally suppresses lens fiber differentiation in the anterior region of lens epithelium by modulating TGFβ and canonical Wnt signaling pathways. These data indicate a novel role of endosome trafficking to ensure equator-dependent commencement of lens fiber differentiation.

## Abstract (precis)

**Summary:** The endocytic regulator VPS45 suppresses FGF-independent lens fiber differentiation and ensures the spatial pattern of lens development.

## INTRODUCTION

In the vertebrate lens, lens epithelium covers the anterior half of the lens fiber core. Lens epithelial cells proliferate and move towards the periphery of the lens epithelium, called the equator (Hanna and O'Brien, 1961). At the equator, lens epithelial cells start to differentiate into lens fiber cells (McAvoy, 1978). Thus, the lens provides a good model for studying spatial regulation of cell differentiation. Fibroblast growth factor (FGF) plays an important role in cell proliferation and differentiation in mammalian lens. Nearly four decades ago it was discovered that co-culturing rat lens epithelial explants with the retina promotes lens fiber cell differentiation, suggesting that secreted molecules emanating from the retina promote lens fiber differentiation (McAvoy, 1980; McAvoy and Fernon, 1984). Later, FGF was identified as a candidate (Chamberlain and McAvoy, 1987, 1989). Importantly, application of FGF to lens epithelium induces cell proliferation at a low dose and lens fiber cell differentiation at a high dose, suggesting that FGF regulates multiple steps of lens fiber differentiation in a dose-dependent manner (McAvoy and Chamberlain, 1989). In mice, lens-specific knockdown of three FGF receptors, FGFR1-FGFR3, severely inhibits lens fiber differentiation (Zhao et al., 2008). Thus, it is believed that fiber differentiation begins at the equator, because the equator is where epithelial cells are first exposed to high levels of FGF that diffuse out of the vitreous body.

In a variety of tissues, canonical Wnt signaling promotes cell proliferation, whereas the non-canonical Wnt/planer cell polarity (PCP) pathway spatially coordinates cell migration and morphogenesis (Niehrs, 2012). Knockdown of a canonical Wnt effector, β-catenin, reduces expression of lens epithelial markers and cell-cycle progression in lens epithelium (Cain et al., 2008). Activation of canonical Wnt signaling displays opposite phenotypes (Martinez et al., 2009), suggesting that canonical Wnt signaling is required for maintenance of lens epithelium. Wnt3a promotes cell-cycle progression in lens epithelial explants, but promotes lens fiber differentiation when explants are pretreated with FGF (Lyu and Joo, 2004). Lens fiber elongation and morphogenesis also require the Wnt/PCP pathway (Chen et al., 2008), which cooperates with FGF signaling (Dawes et al., 2014, 2013). Thus, Wnt signaling regulates lens epithelial cell proliferation and lens fiber cell differentiation in a context-dependent manner.

TGFβ signaling has been implicated in secondary cataracts, also known as posterior capsular opacifications (PCOs). PCOs are the most common complication of cataract surgery and are associated with epithelial-mesenchymal transition (EMT) of lens epithelial cells (Eldred et al., 2011). Injury of mouse lens epithelium, or the anterior capsule, causes EMT through activation of TGFβ signaling, resulting in formation of anterior subcapsular cataracts (ASCs) (Eldred et al., 2011). Application of TGFβ to rat whole lens induces an EMT marker, α-smooth muscle actin (αSMA), and results in ASC-like opaque plaques in lens epithelium (Hales et al., 1995). Thus, TGFβ signaling promotes EMT of lens epithelium. In transgenic mice overexpressing TGFβ1 under control of the lens epithelial cell-specific gene promoter, lens epithelium forms anterior subcapsular plaques, which contain not only αSMA-positive myofibroblastic cells, but also lens fiber-like cells expressing β-crystallin and aquaporin 0 (AQP0; MIPa/b) (Lovicu et al., 2004b), suggesting that high doses of TGFβ induce lens fiber differentiation. Overexpression of dominant-negative TGFβ receptors inhibits lens fiber differentiation in mice (de Iongh et al., 2001), suggesting that TGFβ is required for lens fiber differentiation. However, it is unknown how TGFβ differentially promotes EMT and lens fiber differentiation.

Vacuolar protein sorting 45 (VPS45) belongs to the Sec1/Munc18 (SM) protein family, which promotes assembly of cis-SNARE complexes and subsequent vesicular fusion in yeast (Bryant and James, 2001; Carr and Rizo, 2010) and mammals (Struthers et al., 2009). VPS45 interacts with rabenosyn 5, which functions as a rab5 effector in early endosomes (Nielsen et al., 2000). In *C. elegans* and *Drosophila melanogaster*, VPS45 regulates early endosome formation and further delivery of endocytic cargos from early endosomes to lysosomes (Gengyo-Ando et al., 2007; Morrison et al., 2008). In cultured human cells, VPS45 depletion showed no gross defect in early endosome formation, but it compromised β1-integrin recycling, suggesting that the primary roles of human VPS45 lie downstream of rab5 function in early endosomes, especially in transport of endocytic cargos from early endosomes to recycling endosomes (Rahajeng et al., 2010). Humans with *VPS45* mutations suffer from congenital neutropenia (Vilboux et al., 2013) and lack lysosomes in their fibroblasts (Stepensky et al., 2013), suggesting defects in the endosome-lysosome pathway. Therefore, in humans, VPS45 cooperates with rabenosyn 5 to regulate transport of endocytic cargos from early endosomes to recycling or late endosomes/lysosomes.

In this study, we show that lens epithelial cells abnormally enter fiber differentiation without passing through the equator in zebrafish *vps45* mutants, suggesting that the equator-specific commencement of lens fiber differentiation is affected. Overexpression of *rab5* and *rab11* mRNAs recovered lens phenotypes in *vps45* mutants, suggesting that endocytic trafficking defects cause ectopic lens fiber differentiation. Interestingly, this ectopic lens fiber differentiation does not depend on FGF, but is mediated through activation of TGFβ signaling and suppression of canonical Wnt signaling. Thus, VPS45 normally suppresses lens fiber differentiation in the anterior lens epithelium, by modulating TFGβ and canonical Wnt signaling pathways. These data reveal a novel suppression mechanism of lens fiber differentiation in anterior lens epithelium, which ensures equator-specific onset of lens fiber differentiation.

## RESULTS

### Zebrafish *rw341* mutants show multi-layered lens epithelium

**Fig. 1.**
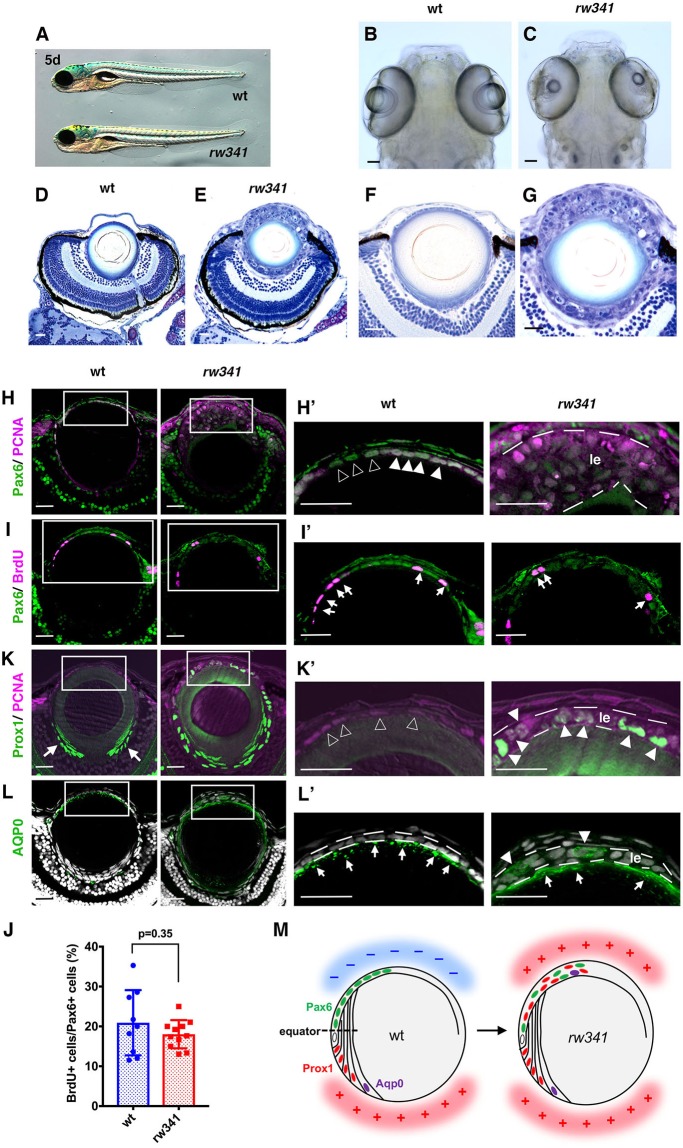
**Ectopic lens fiber differentiation occurs in *rw341* mutant lens epithelium.** (A) Wild-type and *rw341* mutant embryos. (B,C) Wild-type (B) and *rw341* mutant (C) heads. (D,E) Retinas of wild type (D) and *rw341* mutants (E). *rw341* mutants show a small lens fiber core, which is surrounded by many aggregated lens cells. (F,G) Higher magnification of the lenses in D and E. (H,H′) Labeling of lenses using anti-Pax6 and PCNA antibodies. In wild type, lens epithelial cells express Pax6 and PCNA (H′, filled arrowheads). Open arrowheads in H′ indicate cells with relatively weak PCNA expression. In multilayered lens epithelium of *rw341* mutants (H′, le), most cells express PCNA, but Pax6 expression is weak. (I,I′) Labeling of lenses using anti-Pax6 and BrdU antibodies. Arrows indicate BrdU signals. (J) Percentage of BrdU-positive cells among Pax6-positive lens epithelial cells. Data are mean±s.d. No statistical difference between wild type and *rw341* mutants (Student's *t*-test). (K,K′) Labeling of lenses using anti-Prox1 and PCNA antibodies. In wild type, Prox1 is not expressed in lens epithelium (K′, open arrowheads) and only in early differentiating lens fiber cells (K, arrows). In *rw341* mutants, many lens epithelial cells (K′, le) express Prox1 (K′, filled arrowheads). (L,L′) Labeling of lenses using anti-AQP0 antibody. AQP0 is expressed in elongating lens fiber cells in wild type and *rw341* mutants (L′, arrows). AQP0-expressing cells were observed in multilayered *rw341* mutant lens epithelium (L′, arrowheads). (M) Schematic drawing of wild-type and *rw341* mutant lenses. Pax6 is expressed in lens epithelium, whereas Prox1 and AQP0 are expressed in newly differentiating and later elongating lens fiber cells, respectively. In *rw341* mutants, ectopic lens fiber differentiation occurs in multilayered lens epithelium. Areas where lens fiber differentiation occurs and does not occur are indicated by red (+) and blue (−), respectively. All lenses are at 5 dpf. Scale bars: 40 µm in B,C; 20 µm in D-L′.

We screened zebrafish mutants showing defects in lens development, and identified a mutant: *rw341*. At 5 days post-fertilization (dpf), *rw341* mutants showed normal embryonic morphology, except for a lack of expansion of the swim bladder (Fig. 1A); however, mutants have small lens fiber cores (Fig. 1B,C). In wild type, a monolayer of lens epithelium covers the anterior half of a spherical lens fiber core (Fig. 1D,F). However, many cells aggregate to cover the small lens core in *rw341* mutants (Fig. 1E,G). On the other hand, retinal lamination was grossly normal in *rw341* mutants (Fig. 1E), although retinal stem cells located in the ciliary margin were swollen (Fig. S1A). Thus, the monolayer structure of lens epithelium is disrupted in *rw341* mutants. Phalloidin labeling (Fig. S1B) revealed that disruption of lens epithelium appeared between 3 and 4 dpf. Furthermore, lens fiber cells failed to elongate, and aggregated in the posterior region of the lens at 5 dpf (Fig. S1B), suggesting that lens fiber elongation is also compromised in mutants.

### Ectopic lens fiber differentiation occurs in *rw341* mutant lens epithelium

We examined molecular markers of lens epithelial cells at 5 dpf. Zonula occludens 1 (ZO1; Tjp1) is a component of tight and adherens junctions (Fanning and Anderson, 2009), and is observed as dotted signals at the apical interface between lens epithelial cells (Fig. S1C). However, dotted signals were scattered in aggregated anterior lens cells in *rw341* mutants (Fig. S1C). E-cadherin (Shimizu et al., 2005) is located in adherens junctions of lens epithelium. E-cadherin signals were scattered in aggregated anterior lens cells in *rw341* mutants (Fig. S1D). Proliferating cell nuclear antigen (PCNA) is a maker of proliferative cells and is expressed in lens epithelium in zebrafish (Imai et al., 2010). Pax6 is expressed in lens epithelial cells in wild type (Macdonald and Wilson, 1997). In *rw341* mutants, most of the aggregated anterior lens cells expressed PCNA and Pax6, although Pax6 expression was weaker than in wild-type cells (Fig. 1H,H′). Aggregated anterior lens cells therefore maintain lens epithelial fate in the mutant, although the monolayer structure is disrupted.

We next examined cell proliferation. The fraction of BrdU-labeled cells was normally around 20% in lens epithelial cells (Fig. 1I,I′,J). Anti-phosphorylated histone H3 antibody marks mitotic cells, which normally number a few per lens section (Fig. S1E). Fractions of BrdU-labeled and mitotic cells were similar in *rw341* mutants (Fig. 1I,I′,J; Fig. S1E). Thus, it is unlikely that hyper-proliferation disrupts monolayer structure of lens epithelium in *rw341* mutants. As EMT is associated with disruption of monolayered lens epithelium in ASCs (Eldred et al., 2011), we examined expression of an EMT marker αSMA, using a zebrafish transgenic line, *Tg[acta2:EGFP]*, that expresses EGFP under control of the αSMA/acta2 gene promoter (Whitesell et al., 2014). However, we could not detect EGFP expression in *rw341* mutant lens, suggesting that mutant lens epithelial cells are not specified as myofibroblastic cells (Fig. S1F).

Next, we examined markers of lens fiber differentiation. In wild type, Prox1 is expressed in newly differentiating lens fiber cells (Glasgow and Tomarev, 1998; Imai et al., 2010) and is required for lens fiber differentiation (Wigle et al., 1999). An antibody against AQP0 marks elongating lens fiber cells in zebrafish (Froger et al., 2010; Imai et al., 2010). Wild-type anterior lens epithelial cells do not express both Prox1 and AQP0 (Fig. 1K,K′,L,L′). Interestingly, many multilayered anterior lens cells expressed Prox1 (Fig. 1K,K′) in *rw341* mutants at 5 dpf. AQP0-positive cells were also observed in multilayered anterior lens cells, although appeared less often than Prox1-positive cells (Fig. 1L,L′). Thus, lens fiber differentiation occurs abnormally in lens epithelium in *rw341* mutants (Fig. 1M).

To clarify the relationship between lens epithelial disruption and ectopic lens fiber differentiation, we examined a temporal profile of Prox1 and AQP0. In *rw341* mutants at 3 dpf, lens epithelium was monolayered, but Prox1-positive cells appeared (Fig. S2A,B), indicating that ectopic lens fiber differentiation precedes disruption of monolayered lens epithelium. The number of Prox1-positive cells increased progressively until 5 dpf. AQP0-positive cells were observed in *rw341* mutants at 5 dpf, but still at low frequency compared with Prox1-positive cells (Fig. 1L,L′). However, at 9 dpf, most aggregated anterior cells displayed decreased Prox1 expression (Fig. S2C), but increased AQP0 expression (Fig. S2D). Although these AQP0-positive cells did not proceed to denucleation, these data suggest that most Prox1-positive cells enter an AQP0-expressing later stage of lens fiber differentiation by 9 dpf in *rw341* mutants. Thus, in *rw341* mutants, lens epithelial cells undergo lens fiber differentiation without passing through the equator, resulting in disruption of monolayered lens epithelium (Fig. S2E).

### The *rw341* mutant gene encodes VPS45

**Fig. 2.**
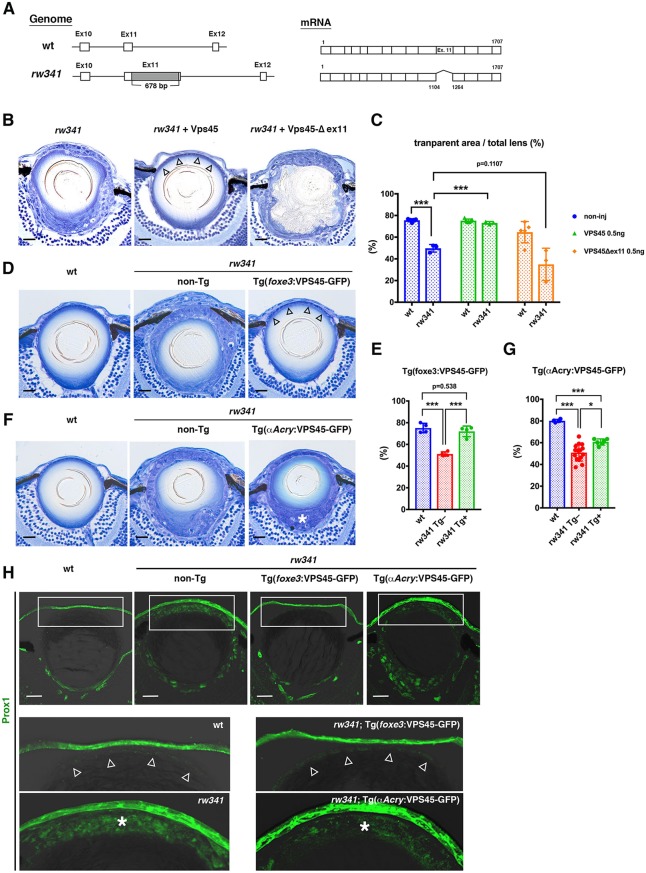
**The *rw341* mutant gene encodes VPS45.** (A) Genomic and cDNA organization of *vps45* gene in *rw341* mutants. (B) 5 dpf lenses of *rw341* mutants (left) and *rw341* mutants injected with wild-type (middle) or mutant (right) *vps45* mRNA. Wild-type *vps45* mRNA rescues multilayer phenotypes (middle, arrowheads). (C) Percentage of transparent lens fiber area in wild type, *rw341* mutants and in *rw341* mutants injected with wild-type or mutant *vps45* mRNA. Wild-type *vps45* mRNA significantly increases transparent area size in *rw341* mutants. (D) 5 dpf lenses of wild type (left), *rw341* mutants (middle) and *rw341* mutants expressing VPS45-GFP under control of the *foxe3* promoter (right). *foxe3* promoter-mediated VPS45-GFP rescues multilayer phenotypes (right, arrowheads). (E) Percentage of transparent lens fiber area in wild type, *rw341* mutants and *rw341* mutants expressing VPS45-GFP under control of the *foxe3* promoter. *foxe3* promoter-mediated VPS45-GFP fully recovers transparent area size in *rw341* mutants. (F) 5 dpf lenses of wild-type (left), *rw341* mutants (middle) and *rw341* mutants expressing VPS45-GFP under control of the *αA-crystallin* promoter (right). Multilayer phenotypes of lens epithelium become milder, but there is still cell aggregation in the posterior lens area in *rw341* mutants overexpressing VPS45-GFP under the *αA-crystallin* promoter (right, asterisk). (G) Percentage of transparent lens fiber area in wild type, *rw341* mutants and *rw341* mutants expressing VPS45-GFP under control of the *αA-crystallin* promoter. *αA-crystallin* promoter-mediated VPS45-GFP partially recovers transparent area size in *rw341* mutants, but the recovery does not reach the wild-type level. (H) Prox1 expression (green) in wild type, *rw341* mutants and *rw341* mutants expressing VPS45-GFP under control of *foxe3* promoter or *αA-crystallin* promoter. Bottom panels indicate higher magnification of outlined areas. Ectopic Prox1 expression in *rw341* mutants is inhibited by *foxe3* promoter-induced VPS45-GFP (arrowheads), but not by *αA-crystallin* promoter-induced VPS45-GFP (asterisks). Fluorescent signals of VPS45-GFP were inactivated by the paraffin sectioning process. (C,E,G) Data are mean±s.d. Two-way (C) and one-way (E,G) ANOVA: *P<0.05, ***P<0.005. Scale bars: 20 µm.

We mapped the *rw341* mutational locus on zebrafish chromosomes, and found that the mutation mapped to the genomic region between two polymorphic markers on chromosome 19 (Fig. S3A, Table S1). The zebrafish genome database revealed that six genes were annotated in the region. We found that *vps45* cDNA lacks exon 11 in *rw341* mutants, probably due to insertion of an unrelated 678 bp sequence into exon 11 of the *vps45* gene (Fig. 2A). No amino acid change was found in the other five genes in *rw341* mutants (data not shown). Next, we injected *vps45* mRNA into *rw341* mutants at the one cell-stage and examined lens phenotypes at 5 dpf. Overexpression of wild-type *vps45* mRNA recovered the monolayer structure of lens epithelium in *rw341* mutants (Fig. 2B) and also the fraction of the transparent area, which corresponds to the lens fiber organelle-free zone (OFZ) (Fig. 2C). On the other hand, overexpression of the mutant *vps45* mRNA that lacks exon 11, did not recover the monolayer structure of lens epithelium and the transparent area (Fig. 2B,C). These data suggest that the *rw341* mutant gene encodes VPS45.

We examined the developmental profile of *vps45* mRNA expression in wild type (Fig. S3B). *vps45* mRNA expression was observed at four-cell and high stages, but disappeared at the shield stage. *vps45* mRNA expression was observed ubiquitously after the tail-bud stage. From 24 hpf to 5 dpf, strong expression was observed in the head region. Plastic sections revealed that *vps45* mRNA was expressed throughout the lens at 24 hpf, but maintained only in lens epithelium at 3 dpf, and only in the peripheral region of lens epithelium, corresponding to the germinative zone, at 5 dpf (Fig. S3C).

To determine which lens epithelial or lens fiber cells require VPS45 functions, we established zebrafish transgenic lines expressing mRNA encoding C-terminal GFP-tagged VPS45 (VPS45-GFP) under the control of *foxe3* (Shi et al., 2006) and *αA-crystallin* (Kurita et al., 2003) promoters, which drive transcription in lens epithelial cells and lens fiber cells, respectively (Fig. S3D,E). First, we confirmed that overexpression of *vps45-GFP* mRNA rescued the *rw341* mutant phenotypes as effectively as *vps45* mRNA (data not shown), suggesting that C-terminal GFP tagging does not affect VPS45 functions. We then combined these transgenes with *rw341* mutants. Overexpression of *vps45-GFP* mRNA by the *foxe3* promoter recovered the monolayer of lens epithelium (Fig. 2D) as well as formation of the transparent lens area in *rw341* mutants (Fig. 2E). On the other hand, overexpression of *vps45-GFP* mRNA by the *αA-crystallin* promoter made the lens epithelial disruption milder in *rw341* mutants (Fig. 2F), but recovery of the transparent lens area was only partial, and less than with *foxe3* promoter-mediated *vps45-GFP* expression (Fig. 2G). Furthermore, overexpression of *vps45-GFP* mRNA by the *foxe3* promoter inhibited ectopic expression of Prox1 in lens epithelium of *rw341* mutants, whereas overexpression of *vps45-GFP* mRNA by the *αA-crystallin* promoter did not (Fig. 2H). Thus, VPS45 activity in lens epithelium is important for inhibition of ectopic lens fiber differentiation.

In *rw341* mutants, loss of exon 11 causes mutant phenotypes. However, loss of exon 11 does not induce a frame shift, but only an internal deletion of 40 amino acids in VPS45 protein. So we examined whether this shortened protein is partially functional. We injected mRNAs encoding VPS45-GFP and C-terminal GFP-tagged VPS45 lacking exon 11 (VPS45-Δex11-GFP) into zebrafish one-cell stage eggs and examined protein stability. GFP expression was detected in both cases at 10 hpf (data not shown). It was observed in 24 hpf embryos injected with *vps45-GFP* mRNA, but not in those injected with *vps45-*Δ*ex11-GFP* mRNA (Fig. S3F). Western blotting with anti-GFP antibody showed that VPS45-Δex11-GFP is absent at 24 hpf (Fig. S3G), suggesting that VPS45-Δex11-GFP is unstable. Next, we examined lens phenotypes in a putative null allele of zebrafish *vps45* mutants, *vps45*^sa14216^, which harbors a nonsense mutation in the ninth position of the 568 amino acid protein (CAG→TAG). We confirmed that all lens phenotypes in *vps45*^sa14216^ are very similar to those of *rw341* mutants (Fig. S4). These data suggest that our *rw341* mutant allele loses most functions of VPS45.

### Endocytic trafficking defects cause *rw341* mutant phenotypes

**Fig. 3.**
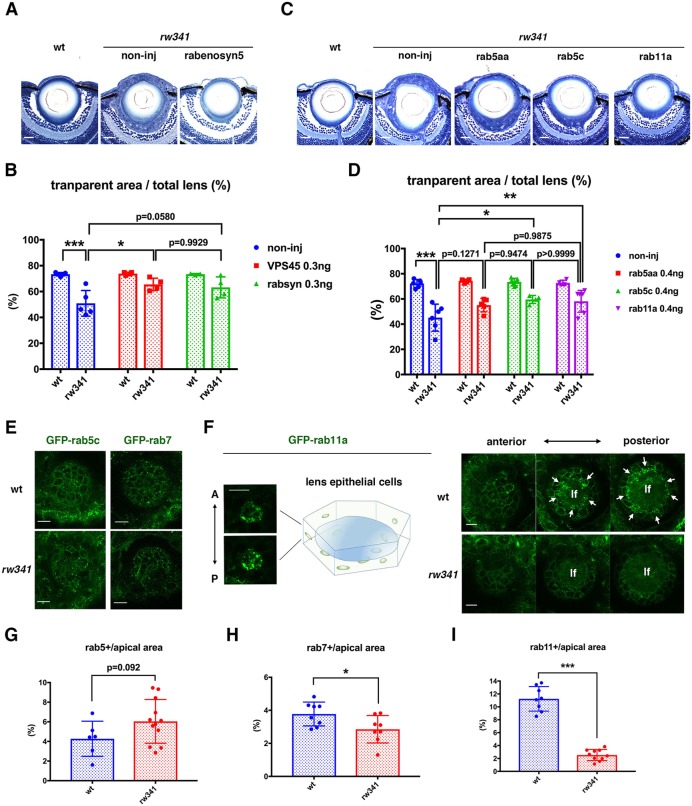
**Endocytic trafficking defects cause *rw341* mutant lens phenotypes.** (A) 5 dpf lenses of wild type, *rw341* mutants and *rw341* mutants injected with *rabenosyn 5* mRNA. (B) Percentage of transparent lens fiber area in wild type, *rw341* mutants and *rw341* mutants injected with *vps45* or *rabenosyn 5* mRNA. *rabenosyn 5* mRNA increased transparent area size in *rw341* mutants, although the difference was less significant (*P*=0.058). (C) 5 dpf lenses of wild type, *rw341* mutants and *rw341* mutants injected with *rab5aa*, *rab5c* or *rab11a* mRNA. (D) Percentage of transparent lens fiber area in wild type, *rw341* mutants and *rw341* mutants injected with *rab5aa*, *rab5c* or *rab11a* mRNA. All types of *rab* mRNA increased transparent area size in *rw341* mutants, although *rab5aa* mRNA injection was not significant. (E) Expression of GFP-tagged rab5c and rab7 in wild-type and *rw341* mutant lens epithelium at 48 hpf. (F) Expression of GFP-tagged rab11a in wild-type and *rw341* mutant lens epithelium at 48 hpf. The left panel provides a schematic drawing of a lens epithelial cell with a nucleus (blue) and recycling endosomes (green). Two different level confocal images indicate GFP-rab11a signals densely located beneath apical surface membranes, which face the lens fiber core (lf) at the posterior-most position along the AP axis. Right panels indicate three neighboring confocal images of wild-type and *rw341* mutant lens epithelium. In wild-type lens, strong patchy GFP-rab11a signals are observed in the apical region of lens epithelial cells (arrows) adjacent to lens fiber core. In contrast, there are no GFP-rab11a signals in *rw341* mutants. (G-I) Percentage of GFP-tagged rab5c (G), rab7 (H) and rab11a (I)-positive area relative to total apical area. (B,D,G-I) Data are mean±s.d. (B,D) Two-way ANOVA. (G-I) Student's *t*-test: *P<0.05, **P<0.01, ***P<0.005. Scale bars: 20 µm.

VPS45 interacts with a rab5 effector, rabenosyn 5, to promote vesicular trafficking along the secretory pathway via early endosomes to recycling and late endosomes (Gengyo-Ando et al., 2007; Morrison et al., 2008; Rahajeng et al., 2010). We injected mRNA encoding mCherry-tagged rab5c and VPS45-GFP into wild-type eggs. In lens epithelium, mCherry-tagged rab5c expression was observed as dotted signals, which correspond to early endosomes (Clark et al., 2011) (Fig. S5A). We observed a weak level of ubiquitous VPS45-GFP expression, as well as strong dotted peaks. VPS45-GFP dotted signals partially overlapped mCherry-rab5c dotted signals. VPS45-GFP and mCherry-rab5c double-positive dots occupied 27.1% of mCherry-rab5c dots (Fig. S5C) and 62.1% of VPS45-GFP dots (Fig. S5D), suggesting that VPS45 colocalizes with rab5c-positive endosomes. However, in *rabenosyn 5* morphant lens epithelium, dotted VPS45-GFP signals were markedly reduced (Fig. S5B). The fraction of double-positive dots in mCherry-rab5c positive dots was reduced to 12.9% in *rabenosyn 5* morphants (Fig. S5C), whereas the fraction of double-positive dots in VPS45-GFP-positive dots was not changed significantly (Fig. S5D). These data suggest that rabenosyn 5 is required for recruitment of VPS45 to rab5-positive early endosomes. Furthermore, either overexpression of *rabenosyn 5*, *rab5aa* or *rab5c* mRNA rescued lens epithelial layer disruption (Fig. 3A,C). *rab5c* mRNA significantly rescued the reduction of the transparent lens area in *rw341* mutants, although its rescue levels by *rabenosyn 5* and *rab5aa* mRNA were partial (Fig. 3B,D). Thus, vesicular trafficking defects via early endosomes are linked to lens phenotypes in *rw341* mutants.

VPS45 is required for early endosome formation in *Drosophila melanogaster* and *C. elegans* (Gengyo-Ando et al., 2007; Morrison et al., 2008). However, formation of early endosomes is normal, but transport of endocytic cargos from early endosomes to recycling endosomes is compromised in VPS45-knockdown human cells (Rahajeng et al., 2010). Furthermore, humans with *VPS45* mutations lack lysosomes in their fibroblasts, suggesting that the maturation process from early to late endosomes is compromised in human *VPS45* mutant cells (Stepensky et al., 2013). We examined the structural integrity of endosomes in *rw341* mutant lens epithelium by expressing RNA encoding GFP-tagged rab5c, rab7 and rab11a, which specifically mark early, late and recycling endosomes, respectively (Clark et al., 2011). GFP-rab5c and GFP-rab7 expression were detected as dotted signals in both wild-type and *rw341* mutant lens epithelium at 2 dpf (Fig. 3E). In wild type, GFP-rab11a expression was observed as dotted signals of variable size that were more densely located in apical regions than in basolateral regions of lens epithelial cells (Fig. 3F). However, GFP-rab11a signals were mostly absent in *rw341* mutants (Fig. 3F). The ratio of GFP-rab5c-positive area to the total apical area was slightly, but not significantly, increased in *rw341* mutants (Fig. 3G). On the other hand, the ratio of GFP-rab7-positive area to the total apical region was decreased in *rw341* mutants (Fig. 3H). Furthermore, the ratio of the GFP-rab11a-positive area relative to the total apical region was drastically decreased in *rw341* mutants (Fig. 3I). Thus, as in human cells, transport of endocytic vesicles from early to late and recycling endosomes is affected in *rw341* mutants. Consistently, overexpression of *rab11a* mRNA significantly rescued lens epithelial defects in *rw341* mutants (Fig. 3C,D).

### *rw341* mutant phenotypes are not related to integrin β1 dysfunctions

It has been reported that lens epithelial disruption and ectopic Prox1 expression occur in *integrin β1*-knockout mice (Simirskii et al., 2007), suggesting that disruption of lens epithelial structures affects the extracellular matrix, which subsequently induces ectopic lens fiber differentiation. However, overexpression of zebrafish *integrin β1a* did not rescue *rw341* mutant lens phenotypes (Fig. S6). Furthermore, electron microscopic analyses revealed that extracellular matrix tissue, which is located along the interface between the three cornea layers (cornea stroma, cornea epithelium and cornea endothelium) and the lens epithelium, was maintained in *rw341* mutants at both 4 and 5 dpf (Fig. S7). Thus, it is unlikely that disruption of extracellular matrix causes ectopic lens fiber differentiation in *rw341* mutants.

### Lens fiber differentiation is independent of FGF signaling in *rw341* mutants

**Fig. 4.**
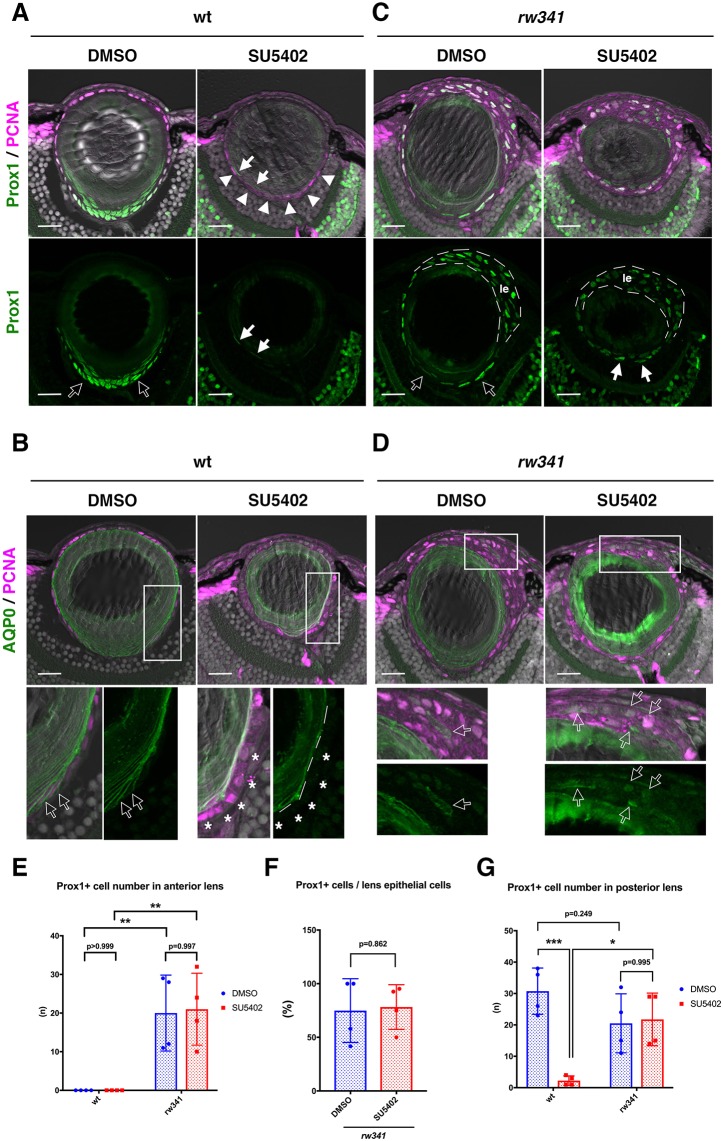
**Ectopic lens fiber differentiation in *rw341* mutants is independent of FGF signaling.** (A) Prox1 and PCNA expression in wild-type lenses. DMSO treatment does not affect PCNA and Prox1 expression, which are normally expressed in lens epithelium and newly differentiating lens fiber cells (open arrows), respectively. SU5402 treatment inhibits Prox1 expression. Almost all cells along the posterior margin of the lens fiber region express only PCNA (arrowheads). A few weakly Prox1-expressing cells are observed inside PCNA-positive posterior marginal cells (filled arrows). (B) AQP0 and PCNA expression in wild-type lenses. Bottom panels indicate higher magnification of outlined areas in the upper panels. DMSO treatment does not affect AQP0 expression. A few peripheral PCNA-positive cells express AQP0 (open arrows). In SU5402 treatment, almost all PCNA-positive posterior marginal cells did not express AQP0 (asterisks). (C) Prox1 and PCNA expression in *rw341* mutant lenses. DMSO treatment does not change Prox1 expression in the posterior lens fiber region (open arrows) as well as the anterior multilayered epithelium (le). SU5402 treatment also did not inhibit Prox1 expression in either the anterior lens epithelium (le) or the posterior lens fiber region (filled arrows). (D) AQP0 and PCNA expression in *rw341* mutant lenses. Bottom panels indicate higher magnification of outlined areas in the upper panels. AQP0-positive cells are detected in the anterior multilayered lens epithelium of both DMSO- and SU5402-treated *rw341* mutants (open arrows). (E) Number of Prox1-positive cells in the anterior lens epithelium per lens section. There are no Prox1-positive cells in wild-type lens epithelium treated with either DMSO or SU5402. SU5402 treatment did not reduce Prox1-positive cells in *rw341* mutants. (F) Percentage of Prox1-positive cells relative to total lens epithelial cells. SU5402 treatment did not reduce the fraction of Prox1-positive cells in *rw341* mutants. (G) Number of Prox1-positive cells in the posterior lens area per lens section. SU5402 treatment drastically reduces Prox1-positive cells in wild type, but did not in *rw341* mutants. (E-G) Data are mean±s.d. (E,G) Two-way ANOVA. (F) Student's *t*-test: *P<0.05, **P<0.01, ***P<0.005. Scale bars: 20 µm (10 µm in higher magnification images in B,D).

FGF signaling is required for lens fiber differentiation in mammals and chicks (Lovicu and McAvoy, 2005; Mochizuki and Masai, 2014). To confirm this inductive role of FGF in zebrafish, we applied an inhibitor of FGF signaling, SU5402, from 2 to 5 dpf. In DMSO-treated wild type, Prox1 is expressed transiently in newly differentiating lens fiber cells (Fig. 4A), whereas AQP0 is expressed in elongating lens fiber cells (Fig. 4B). In SU5402-treated wild type, Prox1 expression was not observed in the posterior lens fiber area (Fig. 4A). Instead, PCNA-positive cells, which are normally observed in lens epithelial cells and newly differentiating lens fiber cells, abnormally occupied the most peripheral region of the posterior lens fiber core in SU5402-treated wild-type embryos (Fig. 4A). All of these PCNA-positive cells were also AQP0 negative (Fig. 4B), but expressed Pax6 (Fig. S8A). Thus, in the absence of FGF signaling, lens epithelial cells fail to enter lens fiber differentiation after passing through the equator. On the other hand, ectopic Prox1 and AQP0 expression were abnormally detected in aggregated anterior lens cells of DMSO-treated *rw341* mutants (Fig. 4C,D). Surprisingly, these anterior Prox1- and AQP0-positive lens cells were still observed in SU5402-treated *rw341* mutants (Fig. 4C-F). These data suggest that ectopic lens fiber differentiation in anterior lens epithelium of *rw341* mutants does not depend on FGF signaling. Furthermore, cells in the most peripheral region of the posterior fiber area expressed PCNA and Prox1 (Fig. 4C), but not Pax6 (Fig. S8B), in SU5402-treated *rw341* mutants. The number of these Prox1-positive cells was similar to that of DMSO-treated *rw341* mutants (Fig. 4G). Thus, equator-dependent lens fiber differentiation also does not depend on FGF signaling in *rw341* mutants.

There are two possibilities that explain the lack of dependency of lens fiber differentiation on FGF signaling in *rw341* mutants. The first is that lens epithelial cells undergo an FGF-independent bypass pathway for lens fiber differentiation in *rw341* mutants. The second possibility is that either the level or sensitivity of FGF ligand is enhanced in *rw341* mutants; the SU5402 treatment is not enough to prevent stimulation of the FGF signaling pathway below the threshold that triggers lens fiber differentiation. To explore these two possibilities, we examined expression of an FGF target gene, *pea3* (Brown et al., 1998). In wild type, *pea3* mRNA was expressed in the equatorial region (Fig. S9), suggesting that FGF signaling is linked to equator-specific commencement of lens fiber differentiation. In *rw341* mutants, the spatial pattern of *pea3* mRNA expression did not differ from that of wild type. In particular, there was no *pea3* expression in anterior lens epithelium of *rw341* mutants (Fig. S9). These data support the first possibility: that lens epithelial cells employ an FGF-independent pathway for lens fiber differentiation in *rw341* mutants.

### Ectopic lens fiber differentiation depends on TGFβ signaling in *rw341* mutants

**Fig. 5.**
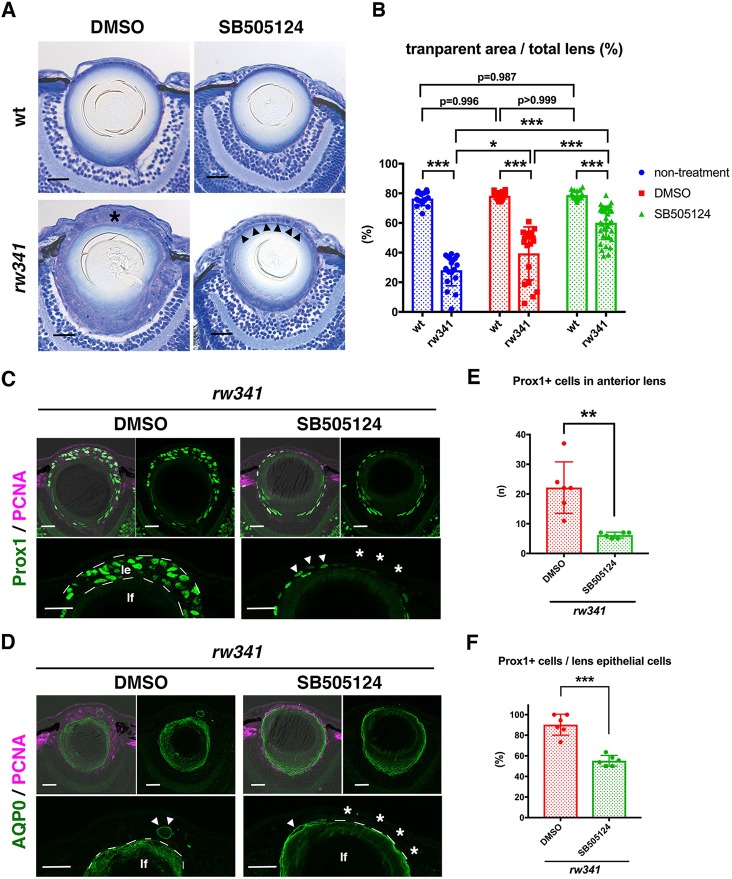
**Ectopic lens fiber differentiation depends on TGFβ signaling.** (A) 5 dpf wild-type and *rw341* mutant lenses treated with DMSO and SB505124. In DMSO-treated *rw341* mutants, lens epithelial cells form multilayers (asterisk). SB505124 treatment rescues multilayer phenotypes in *rw341* mutants (arrowheads). (B) Percentage of transparent lens fiber area in wild type and *rw341* mutants treated with DMSO and SB505124. SB505124 treatment significantly increases transparent fiber area size in *rw341* mutants, although the recovery does not reach the wild-type level. (C) Prox1 (green) and PCNA (magenta) expression in 5 dpf *rw341* mutant lenses treated with DMSO and SB505124. Lower panels indicate higher magnification of lens epithelia (le). lf, lens fiber. Upper right and lower panels show only the green channel. In DMSO-treated *rw341* mutants, most cells express Prox1 in multilayered lens epithelium (le). In BS505124-treated *rw341* mutants, the number of Prox1-positive cells is markedly reduced (arrowheads). Asterisks indicate the Prox1-negative lens epithelial area. (D) AQP0 (green) and PCNA (magenta) expression in 5 dpf *rw341* mutant lenses treated with DMSO and SB505124. Lower panels indicate higher magnification of lens epithelia. Upper right and lower panels show only the green channel. In DMSO-treated *rw341* mutants, AQP0-positive lentoid-like structures are observed (arrowheads). In SB505124-treated *rw341* mutants, only one PCNA-positive cell expresses AQP0 (arrowhead), but lentoid-like structure is not observed (asterisks). (E) The number of Prox1-positive cells in anterior lens epithelium per lens section. (F) Percentage of Prox1-positive cells relative to the total number of lens epithelial cells. (B,E,F) Data are mean±s.d. (B) Two-way ANOVA. (E,F) Student's *t*-test: *P<0.05, **P<0.01, ***P<0.005. Scale bars: 20 µm.

TGFβ induces EMT of lens epithelium (Eldred et al., 2011). Furthermore, transgenic mice expressing TGFβ1 under the control of a lens epithelium-specific promoter induces not only EMT, but also lens fiber differentiation (Lovicu et al., 2004b). Accordingly, we examined TGFβ signaling. Binding of TGFβ to its receptors induces phosphorylation of Smad2 (Moustakas and Heldin, 2009). Ubiquitous weak signals of phosphorylated Smad2 with a few dotted strong peaks were observed in nuclei of wild-type lens epithelial cells (Fig. S10A). However, phosphorylated Smad2 signals were stronger and more broadly observed in lens epithelial nuclei of *rw341* mutants (Fig. S10A), suggesting that TGFβ signaling is enhanced. Next, we applied an inhibitor of TGFβ signaling, SB505125 (DaCosta Byfield et al., 2004). After SB505125 treatment from 1 dpf, dot-like signals of phosphorylated Smad2 disappeared in wild-type lens epithelium, and phosphorylated Smad2 levels decreased markedly in *rw341* mutant lens epithelium at 5 dpf (Fig. S10B), confirming that SB505125 inhibited TGFβ signaling. Interestingly, SB505125 treatment significantly suppressed lens epithelial disruption (Fig. 5A) and recovered the transparent lens fiber area in *rw341* mutants at 5 dpf (Fig. 5B). Furthermore, ectopic Prox1 and AQP0 expression was reduced in anterior lens epithelium of *rw341* mutant embryos treated with SB505125 (Fig. 5C,D). In *rw341* mutants treated with SB505125, the number of Prox1-positive cells in the anterior lens epithelium decreased drastically (Fig. 5E). The percentage of Prox1-positive cells, relative to total lens epithelial cells, also decreased from 90.2% to 55.2% (Fig. 5F). Thus, ectopic lens fiber differentiation depends on TGFβ signaling in *rw341* mutants.

### Wnt activation suppresses ectopic lens fiber differentiation in *rw341* mutants

**Fig. 6.**
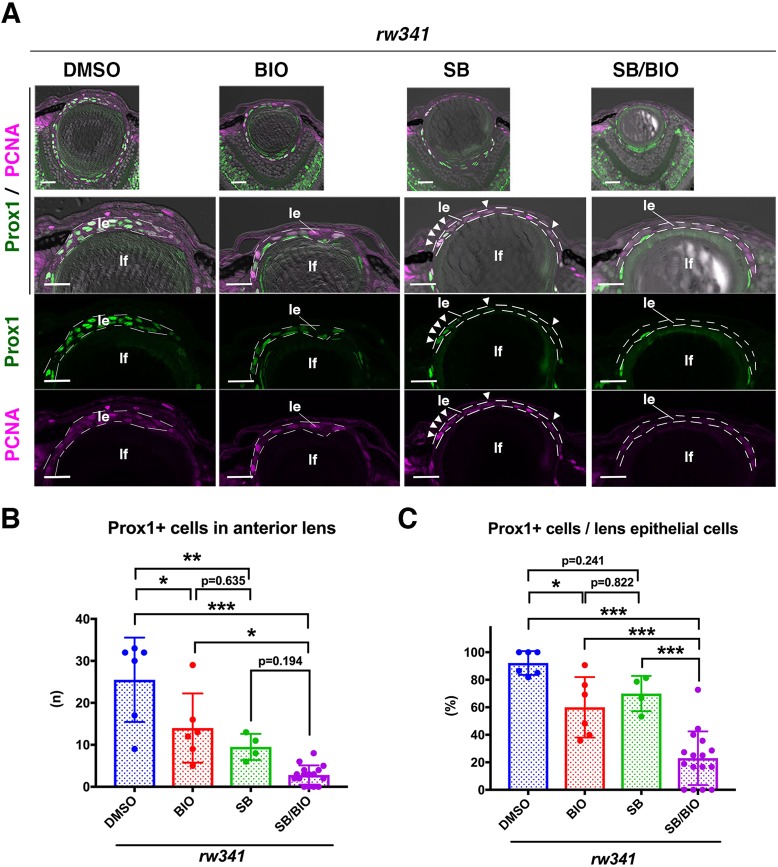
**Wnt activation rescues ectopic lens fiber differentiation in *rw341* mutants.** (A) Prox1 (green) and PCNA (magenta) expression in 5 dpf *rw341* mutant lenses treated with DMSO, BIO, SB505124 and SB505124/BIO. The second row provides higher magnification of lens epithelia (le). Third and fourth rows show only green and magenta channels, respectively. In BIO-treated *rw341* mutant lenses, lens epithelial cells still form multilayers and express Prox1. In SB505124-treated *rw341* mutants, lens epithelium is monolayered and the number of Prox1-positive cells is reduced (arrowheads). In *rw341* mutants treated with SB505124 and BIO, lens epithelium is monolayered and almost all lens epithelial cells are Prox1 negative. lf, lens fiber. (B) The number of Prox1-positive cells in lens epithelium of *rw341* mutants per lens section. Either BIO or SB505124 treatment reduced the number of Prox1-positive cells. Dual treatment with SB505124 and BIO more effectively reduced the number of Prox1-positive cells in *rw341* mutants than BIO treatment. However, no significant difference between SB505124 and BIO/SB505124 treatments may indicate that effective rescue of monolayered epithelial structure by SB505124 is enough to reduce ectopic Prox1 cell number. (C) Percentage of Prox1-positive cells in anterior lens epithelium of *rw341* mutants. BIO treatment significantly reduced the percentage, which is similar to the outcome of SB505124 treatment. Dual treatment with SB505124 and BIO more effectively reduced the percentage of Prox1-positive cells than either SB505124 or BIO treatment alone. (B,C) Data are mean±s.d. One-way ANOVA: *P<0.05, **P<0.01, ***P<0.005. Scale bars: 20 µm.

**Fig. 7.**
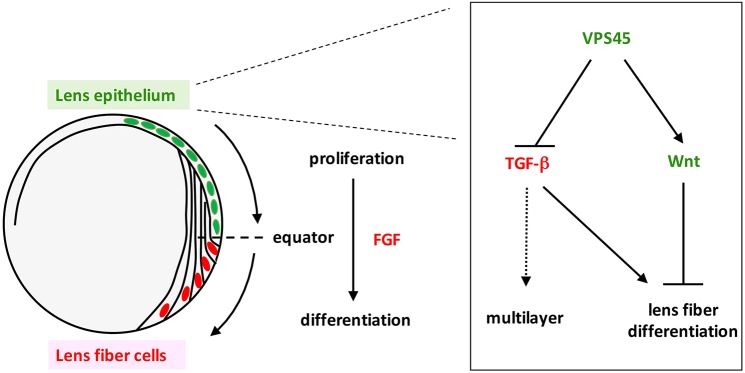
**A novel suppression mechanism for lens fiber differentiation.** FGF signaling initiates lens fiber differentiation at the equator. VPS45 normally suppresses lens fiber differentiation in lens epithelium by suppressing TGFβ signaling and maintaining canonical Wnt signaling. Multilayered epithelial phenotypes result from ectopic lens fiber differentiation or may be directly induced by TGFβ signaling. This novel VPS45-mediated suppression mechanism ensures equator-specific onset of lens fiber differentiation.

The blockade of TGFβ signaling markedly rescued lens epithelial disruption in *rw341* mutants (Fig. 5A), but more than 50% of lens epithelial cells still expressed Prox1 (Fig. 5C,F). This mild rescue of ectopic Prox1 expression suggests that other factors promote ectopic lens fiber differentiation. Canonical Wnt signaling promotes maintenance of lens epithelium in mice (Cain et al., 2008; Martinez et al., 2009). Thus, we examined whether canonical Wnt signaling is inactivated in lens epithelium of *rw341* mutants. We crossed wild-type and *rw341* mutant individuals with a TOP:dGFP transgenic line that carries dGFP under control of the β-catenin-responsive promoter (Dorsky et al., 2002). In this transgenic line, TOP:dGFP mRNA was expressed in lens epithelium (Fig. S11A) and GFP fluorescence was detected after lens epithelial cells enter lens fiber differentiation, probably owing to the time lag for GFP protein translation and maturation (Fig. S11B). We found that the TOP:dGFP signal was not observed in either multilayered anterior lens cells or the posterior lens fiber area in *rw341* mutants (Fig. S11B), suggesting that canonical Wnt signaling is suppressed in lens epithelium of *rw341* mutants. Next, we applied a Wnt activator, BIO, to *rw341* mutants. BIO treatment did not rescue lens epithelial disruption (Fig. 6A), but decreased the number of Prox1-positive cells (Fig. 6B). The percentage of Prox1-positive cells in lens epithelium of *rw341* mutants was reduced to 60%, suggesting an intermediate suppression of lens fiber differentiation, similar to that seen with SB505124 treatment (Fig. 6C). Interestingly, double treatment of *rw341* mutants with BIO and SB505124 rescued multilayer phenotypes (Fig. 6A) and more effectively reduced the percentage of Prox1-positive cells in lens epithelium than either BIO or SB505124 alone (Fig. 6C). These data suggest that ectopic Prox1 expression is induced by both activation of TGFβ signaling and inhibition of canonical Wnt signaling. We confirmed that monolayered lens epithelium expressed Pax6 in SB505124/BIO-treated *rw341* mutants (Fig. S12). Taken together, these data suggest that VPS45 normally suppresses TGFβ signaling and maintains canonical Wnt signaling in anterior lens epithelium, resulting in suppression of ectopic lens fiber differentiation (Fig. 7).

## DISCUSSION

In this study, we have identified a zebrafish *vps45* mutation, which disrupted the monolayer structure of lens epithelium and induced ectopic lens fiber differentiation without passing through the equator. Previously, similar lens epithelial disruption and ectopic fiber differentiation were observed in integrin β1 mutant mice (Simirskii et al., 2007), suggesting that disruption of lens epithelium or extracellular matrix tissue induces ectopic lens fiber differentiation. However, overexpression of integrin β1 did not rescue lens phenotypes in *vps45* mutants. Second, ectopic fiber differentiation occurs in *vps45* mutants prior to disruption of epithelial monolayer structure. Third, extracellular matrix tissue of the lens capsule is maintained in *vps45* mutants. Thus, ectopic lens fiber differentiation is not primarily caused by disruption of lens epithelium, but more directly by the loss of VPS45 activity. In vertebrates, FGF is essential for lens fiber differentiation (Chamberlain and McAvoy, 1987, 1989; Zhao et al., 2008). Surprisingly, ectopic lens fiber differentiation in *vps45* mutants does not depend on FGF signaling, but is associated with activation of TGFβ signaling and inactivation of canonical Wnt signaling. Thus, VPS45 normally suppresses lens fiber differentiation in anterior lens epithelium by modulating TGFβ and canonical Wnt signaling pathways in an FGF-independent manner. This finding is very interesting because lens epithelium has a novel mechanism for repressing lens fiber differentiation, which ensures equator-specific onset of lens fiber differentiation.

In *C. elegans* and *Drosophila melanogaster*, VPS45 cooperates with rabenosyn 5 to promote early endosome formation (Gengyo-Ando et al., 2007; Morrison et al., 2008). However, in humans, VPS45 is not required for rab5-mediated early endosome formation, but regulates further delivery of endocytic cargos from early to recycling endosomes (Rahajeng et al., 2010). Furthermore, humans carrying a mutation in *VPS45* gene lack lysosomes in their fibroblasts (Stepensky et al., 2013), suggesting that VPS45 regulates maturation steps of early endosomes into late endosomes/lysosomes. We found that early endosomes are maintained in lens epithelium of *vps45* mutants; however, late and recycling endosomes decrease significantly. Thus, lens phenotypes in *vps45* mutants are caused by defects in endocytic trafficking, through the pathway from early endosomes to recycling and late endosomes.

How does VPS45 knockdown activate TGFβ signaling and inhibit canonical Wnt signaling? Endocytosis plays dynamic roles in modulating signaling transduction (Dobrowolski and De Robertis, 2011). After a TGFβ ligand-receptor complex is formed in plasma membrane, the ligand-receptor complex is internalized and transported to early endosomes, where Smad anchor for receptor activation (SARA) recruits Smad2, after which the TGFβ ligand-receptor complex phosphorylates Smad2 (Tsukazaki et al., 1998). In this context, trafficking of the TGFβ ligand-receptor complex to early endosomes is required for activation of TGFβ signaling. Indeed, this idea is supported by a previous report that rab5 knockdown compromises Nodal signaling in zebrafish (Kenyon et al., 2015). Reduction of recycling and late endosomes in *vps45* mutant may compromise delivery of endocytic cargos from early endosomes to late or recycling endosomes. In this case, the TGFβ ligand-receptor complex may be stably retained in early endosomes, leading to prolonged activation of TGFβ signaling.

In the absence of Wnt ligands, β-catenin is effectively degraded by the destruction complex, in which β-catenin is phosphorylated by glycogen synthase kinase 3β (GSK3β). Binding of Wnt ligands to their receptors, Frizzled and Lrp5/6, triggers recruitment of Dvl, Axin and GSK3β to the plasma membrane, leading to formation of Lrp6-positive signalosomes (Bilic et al., 2007). Lrp6-positive signalosome formation inhibits GSK3β-mediated phosphorylation of β-catenin, resulting in stabilization of cytoplasmic β-catenin (Metcalfe and Bienz, 2011). Recently, another model linked to endocytic trafficking was proposed. In this model, Lrp6-positive signalosomes are internalized by endocytosis and transported to late endosomes, where GSK3β is transported to intraluminal vesicles of multivesicular endosomes, resulting in sequestration of cytoplasmic GSK3β (Dobrowolski et al., 2012; Taelman et al., 2010). Reduction of cytoplasmic GSK3β enables newly synthesized β-catenin to accumulate, translocate into the nucleus and activate downstream targets. In this scenario, trafficking of Lrp6 signalosomes to late endosomes is essential for sustained active canonical Wnt signaling. Reduction of late endosomes in *vps45* mutants may compromise lysosome-mediated GSK3β sequestration. In addition, a Wnt inhibitor, Dkk1, removes Lrp6 from lipid rafts and promotes its internalization (Sakane et al., 2010). In this case, Lrp6 is subsequently trafficked on a rab7-dependent route to lysosomes or in a rab11-dependent recycling route to the plasma membrane. The arrest of endocytic trafficking to recycling endosomes in *vps45* mutants may compromise Lrp6 maintenance, resulting in constitutive inhibition of canonical Wnt signaling. Although further investigation is required, VPS45 is clearly an important modulator of these signaling pathways.

Next, how do activated TGFβ signaling and inactivated canonical Wnt signaling induce lens fiber differentiation? In mice, β-catenin knockdown reduced expression of lens epithelial markers, such as Pax6 (Cain et al., 2008). It has been reported that small eye alleles of *Pax6* mutant mice, *Pax6*^Sey^ in heterozygotes show the formation of anterior subcapsular plaques, which express both EMT markers and lens fiber markers similar to transgenic mice overexpressing TGFβ (Lovicu et al., 2004b), suggesting that a decreased level of Pax6 promotes lens fiber differentiation. Indeed, Pax6 expression in anterior lens epithelium is weaker in *vps45* mutants than in wild type. Thus, reduction of canonical Wnt signaling may facilitate lens fiber cell differentiation through downregulation of Pax6.

TGFβ signaling has been implicated in ASCs and secondary cataracts known as PCOs (Eldred et al., 2011), in which the lens epithelial cells retained in the lens capsule migrate like EMT, and become myofibroblastic cells and, in some case, lentoid-like cells called Elschnig's pearl. Thus, TGFβ promotes fibrous type ASC (Saika, 2004) and PCO (Hales et al., 1995). However, little is known about mechanism of lens fiber-type ASC and PCO. Overexpression of constitutive active TGFβ under control of the *α-crystallin* promoter induces ASCs, which express both EMT and lens fiber differentiation markers in mice (Lovicu et al., 2004a,b), suggesting that excessive TGFβ induces ectopic lens fiber differentiation in lens epithelial cells. TGFβ signaling is mediated by Smad-dependent and -independent pathways (Moustakas and Heldin, 2009), the latter leading to activation of ERK-MAPK (Lee et al., 2007), p38-MAPK (Galliher and Schiemann, 2007) and JNK signaling cascades (Sorrentino et al., 2008). Smad3 knockdown reduced, but did not completely inhibit, TGFβ-induced EMT and lens fiber differentiation (Banh et al., 2006; Robertson et al., 2007), suggesting that the Smad3-independent pathway mediates TGFβ-induced ectopic lens fiber differentiation. It has been reported that inhibition of the mTOR pathway effectively suppresses TGFβ-induced lentoid formation in *in vitro* cultures reconstructed from dissociated embryonic chick lens epithelial cells (Boswell et al., 2017), suggesting that the PI3K-Akt-mTOR pathway is involved in TGFβ-mediated lens fiber differentiation. It will be interesting to determine whether the Smad3-independent pathway and PI3K-Akt-mTOR pathway are altered in *vps45* mutants. Further studies on the downstream pathway in *vps45* mutants will advance understanding of spatial regulation of lens fiber differentiation and provide insight into the pathological process of secondary cataracts.

## MATERIALS AND METHODS

### Fish strains

Zebrafish (*Danio rerio*) were maintained in accordance with standard procedures (Westerfield, 1993). RIKEN wako (RW) and WIK were used as wild-type strains for mutagenesis and mapping of the *rw341* mutational locus, respectively. General analyses were carried out using the *rw341* mutant line maintained in the genetic background of Okinawa wild type (oki). The severity of lens phenotypes in *rw341* mutants was similar between in RW, WIK and oki genetic backgrounds. Two *vps45* mutant alleles, *vps45*^rw341^ and *vps45*^sa14216^, were used. *vps45*^sa14216^ was provided by the Zebrafish International Resource Center (ZIRC, Eugene, OR, USA). Transgenic lines *Tg[foxe3:VPS45-GFP]*^oki041^ and *Tg[αAcy:VPS45-GFP]*^oki039^ were used. *Tg[TOP:dGFP]*^w25^ was used to monitor the activation of canonical Wnt signaling (Dorsky et al., 2002) and *Tg[acta2:EGFP]^ca7^* was used to evaluate αSMA expression (Whitesell et al., 2014).

### Ethics statement

All zebrafish experiments were performed in accordance with the Animal Care and Use Program of Okinawa Institute of Science and Technology Graduate School (OIST), Japan, which is based on the Guide for the Care and Use of Laboratory Animals by the National Research Council of the National Academies and has been accredited by the Association for Assessment and Accreditation of Laboratory Animal Care (AAALAC International). All the experimental protocols were approved by the OIST Institutional Animal Care and Use Committee.

### Mutagenesis and cloning of the mutant gene

*rw341* mutants were identified by screening ocular morphology-defective mutants. Mutagenesis, mapping and cloning procedures were carried out as previously described (Masai et al., 2003). Information on polymorphic markers used for mapping is shown in Table S1.

### Histology

Plastic sectioning, immunolabeling of cryosections and paraffin sections, *in situ* hybridization of RNA probes, and EM analyses were performed as described previously (Imai et al., 2010). BrdU incorporation was carried out in accordance with our previous studies (Yamaguchi et al., 2005). Nuclear staining was performed using 50 nM SYTOX Green (Molecular Probes) or 1 nM TOPRO3 (Molecular Probes). Images were scanned using a confocal laser scanning microscope FV1000 (Olympus) and LSM510Mata/LSM710 (Carl Zeiss).

### Antibodies

Anti-Cdh1 (E-cadherin) antibody (Gene Tex, GTX125890, 1:200) was applied to cryosections. Antibodies against BrdU (BioRad, MCA2060, 1:200), phosphorylated HistoneH3 (upstate, 06-570, 1:100) and ZO-1 (Invitrogen, 33-9100, 1:100) were applied to cryosections pretreated at 120°C for 20 min in 10 mM citrate buffer (pH 6.0). Antibodies against PCNA (clone PC10, Sigma P8825; 1:200), Pax6 (BioLegend, PRB-278P, 1:200-1:500), AQP0 (Millipore, AB3071, 1:200-1:500), Prox1 (Millipore, AB5475, 1:200 or Gene Tex, GTX128354, 1:500) and p-Smad2 (Cell Signaling, 3101S, 1:100) were applied to paraffin sections pretreated with heat [120°C for 20 min, in 10 mM citrate buffer (pH 6.0)].

### DNA construction and RNA injection

To induce expression of zebrafish *vps45*, *vps45*Δ*ex11*, *rabenosyn 5*, *rab5aa*, *rab5c*, *rab11a* and *integrin β1* mRNAs, their coding regions were subcloned into the pCS2 expression vector. A GFP-tag was added to the N termini of *rabenosyn 5*, *rab5aa, rab5c* and *rab11a* or to the C-termini of *vps45* and *vps45*Δ*ex11* cDNA. Their linearized plasmids were used to synthesize mRNA using a mMESSAGE mMACHINE SP6 kit (Ambion). mRNA (1 nl) encoding VPS45-GFP (300-500 µg/ml), VPS45Δex11-GFP (500 µg/ml), integrin β1 (300-1200 µg/ml), GFP-rabenosyn 5 (300 µg/ml), GFP-rab5aa (400 µg/ml), GFP-rab5c (400 µg/ml) and GFP-rab11a (400 µg/ml) was injected into fertilized eggs. Western blotting of *vps45-GFP* and *vps45*Δ*ex11-GFP* mRNA injected embryos with anti-GFP antibody (Invitrogen, A11122; 1:500) were carried out as described previously (Imai et al., 2010). Information on genes we used in this study are shown in Table S2.

### Colocalization of Rab5 and VPS45 in the lens epithelium

A pCS2 expression vector-containing N-terminal mCherry-tagged *rab5c* and *VPS45-GFP* were prepared and used for synthesis of mRNA using a mMESSAGE mMACHINE SP6 kit (Ambion). ATG-morpholino against *rabenosyn 5* (MO-*rabenosyn 5*) and standard MO were designed as 5′-GGCCATCGGCTACAGAGAACTACTG-3′ and 5′-CCTCTTACCTCAGTTACAATTTATA-3′, respectively. Either MO-*rabenosyn 5* or standard MO were co-injected into zebrafish eggs at 500 µM with a mixture of *mCherry-rab5c* and *vps45-GFP* mRNA (each 50 µg/ml). mCherry-rab5c and VPS45-GFP signals in lens epithelium at 50 hpf were scanned using a confocal LSM510 microscope (Carl Zeiss). Using the surface rendering tool of Imaris software (Ver. 8.2.1), red and green surface objects were created, representing mCherry-rab5c- and VPS45-GFP-positive dotted signals, respectively (bottom panels of Fig. S5A,B). Numbers of mCherry-rab5c-positive dots, VPS45-GFP-positive dots and mCherry-rab5c/VPS45-GFP double-positive dots were examined by counting the number of red, green and red/green overlapping objects, respectively. The fraction of double-positive dots was calculated in mCherry-rab5c and VPS45-GFP-positive dot populations.

### Evaluation of early, late and recycling endosomes in lens epithelial cells

pCS2 expression vector-containing N-terminal GFP-tagged *rab5c*, *rab7* and *rab11a* were prepared and used for synthesis of mRNA using a mMESSAGE mMACHINE SP6 kit (Ambion). mRNA encoding GFP-rab5c, rab7 and rab11a (1 nl of 100 µg/ml each) was injected at the one-cell stage into *rw341* mutant and wild-type sibling eggs, which were produced by a pair of *rw341* heterozygous fish. Lens epithelium was scanned with confocal microscopy at 48 hpf for more than 20 embryos for each RNA injection. After confocal scanning, DNA was extracted from each scanned embryo, and used for genotyping. Using scanned images, one to three lens epithelial cells, the apical regions of which were clearly scanned, were selected from each wild-type and *rw341* mutant embryo, and used for measurement of total apical area size and GFP-positive area using ImageJ (NIH).

### Measurement of transparent lens fiber area

Plastic sections of 5 dpf lenses were labeled with Toluidine Blue, which visualizes the boundary between peripheral lens nucleated cells (lens epithelium and elongating lens fiber cells) and central transparent lens fiber area. Central transparent lens fiber area corresponds to the lens OFZ, where intracellular organelles, such as nuclei and mitochondria, are eliminated (Imai et al., 2010). Using the software, ImageJ (NIH), total lens and transparent fiber areas were measured in each lens section. The ratio of transparent fiber area relative to total lens area was examined for each lens, and the average ratio was calculated for each genetic combination, using more than three lenses from different individuals.

### Establishment of transgenic lines: *Tg[foxe3:VPS45-GFP]* and *Tg[αAcy:VPS45-GFP]*

We cloned the *foxe3* promoter, which covers a 7 kb genomic region upstream of the *foxe3*-coding region, and confirmed that this promoter specifically drives lens epithelium-specific transcription in zebrafish, using GFP as a reporter (data not shown). This 7 kb *foxe3* promoter and the 2.5 kb *αA-crystallin* promoter (Kurita et al., 2003) were inserted between the *Xho*I and *Bam*HI sites of the Tol2-base expression vector, pT2AL200R150G (Urasaki et al., 2006), respectively. DNA fragment encoding VPS45-GFP was further inserted between *Bam*HI and *Cla*I sites of pT2AL200R150G to fuse these promoters. These plasmids were injected into fertilized eggs. Injected F0 fish were raised to the adult stage and used for establishment of transgenic lines from F1 progeny. After we established these transgenic lines, we confirmed that VPS45-GFP was expressed exclusively in lens epithelium and lens fiber cells in *Tg[foxe3:VPS45-GFP]* and *Tg[αAcy:VPS45-GFP]*, respectively (Fig. S3D,E).

### Chemical treatment

The chemical inhibitors SB505124 (S4696, Sigma), SU5402 (572630, Calbiochem) and BIO (B1689, Sigma) were dissolved in E3 medium (5 mM NaCl, 0.17 mM KCl, 0.4 mM CaCl_2_ and 0.16 mM MgSO_4_) containing 1% DMSO at 50 µM, 5 µM and 1.25 µM, respectively. SB505124 was applied from 24 hpf. SU5402 and BIO were applied from 48 hpf. Treated embryos were fixed with 4% paraformaldehyde (PFA) at 120 hpf.

### Sample size and statistical analyses

The numbers of embryos or cells used for statistical analyses are shown in Table S3. Averages and standard deviations were calculated. Using the statistical package R (CRAN, ver. 3.5.0.), a normal distribution of data in each group was confirmed using a one-sample Kolmogorov–Smirnov test (two sided) (data not shown). Homogeneity of variance was estimated with an *F*-test, and variance was estimated to be equal in 52 of total 66 group combinations, but the remaining 24 combinations were non-equal (data not shown). Thus, statistical analyses were performed using Student's *t*-tests (two-sided test; unequal variance, Welch correction) or one- or two-way ANOVA (multiple comparison, Tukey) (Table S3). To confirm that sample size was appropriate, we measured post hoc-achieved power for all samples showing statistical significance (α error probability, *P*<0.05), using a free software G*Power 3.1.9.3 (University of Düsseldorf, Heinrich Heine) (Table S4). In 45 of the 50 sample combinations, power was >0.8. The remaining five samples showed a range of power from 0.5 to 0.8. To keep statistical variability caused by genetic background variation to a minimum, we used siblings generated from the same parent fish for comparison between experimental and control groups. After genotyping embryos, a sufficient number of experimental and control samples was selected at random from the same siblings. Once we examined selected samples, all samples were used for statistical analyses.

## Supplementary Material

Supplementary information
