# Supplementary material for: Endocytic trafficking factor VPS45 is essential for spatial regulation of lens fiber differentiation in zebrafish
Source: Development. 2018 Oct 15;145(20):dev170282. doi: 10.1242/dev.170282 (PMC6215396; doi:10.1242/dev.170282)
Supplement: Supplementary information [file develop-145-170282-s1.pdf]

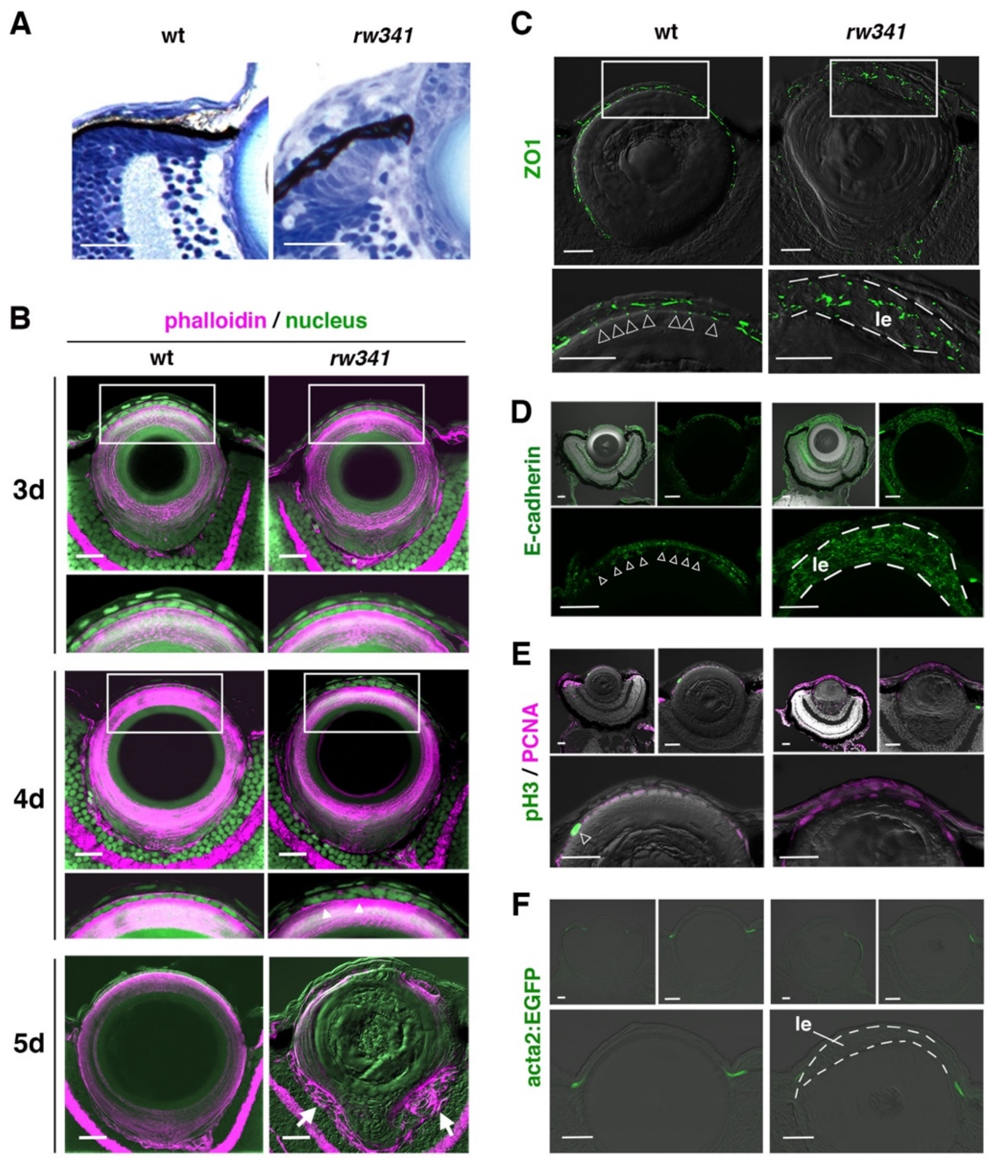

***Figure S1*: Lens epithelial phenotypes of the *rw341* mutant**

(A) Retinal ciliary marginal zone (CMZ) of wild-type and *rw341* mutant embryos at 5 dpf. Retinal stem cells and progenitor cells, which are located in the retinal CMZ, were swollen in *rw341* mutants.

(B) Labeling of wild-type and *rw341* mutant lenses with phalloidin (magenta) and a nuclear stain, Sytox-Green (green) at 3, 4, and 5 dpf. Higher-magnification images of squares in upper panels are shown at the bottom of each panel. In *rw341* mutants, lens epithelium is monolayered at 3 dpf, but lens epithelial nuclei become swollen and start to pile up (arrowheads) at 4 dpf. Irregularly aggregated cells (arrows) are observed in the posterior region of the lens in *rw341* mutants at 5 dpf.

(C) Labeling 5 dpf wild-type and *rw341* mutant lenses with anti-ZO1 antibody (green). Upper and lower panels indicate a lens and higher magnification of lens epithelium, respectively. Dot-like ZO1 signals are detected at the apical interface between wild-type lens epithelial cells (open arrowheads), whereas dotted ZO1 signals are irregularly observed in multilayered lens epithelium of *rw341* mutants (le).

(D) Labeling of 5 dpf wild-type and *rw341* mutant lenses with antibodies against E-cadherin (green). Nuclei were stained with TOPRO3 (white). Upper left and right panels indicate an eye and a lens, respectively. Lower panels indicate higher magnification of the lens epithelium area. Right upper and lower panels indicate only the green channel. E-cadherin is normally localized at the adherens junction between wild-type lens epithelial cells (open arrowheads), but scattered in the multilayered lens epithelium of *rw341* mutants (le).

(E) Labeling of 5 dpf wild-type and *rw341* mutant lenses with anti-pH3 (green) and anti-PCNA (magenta) antibodies. Nuclei were counter-stained with TOPRO3 (white). Upper left and right panels indicate an eye and a lens, respectively. Lower panels indicate higher magnification of lens epithelium area. pH3-positive lens epithelial cells are few in wild type (open arrowhead) and do not increase in *rw341* mutants.

(F) Confocal scanning of 5 dpf wild-type and *rw341* mutant lenses with a transgene, *Tg[acta2: EGFP]* (green). Upper left and right panels indicate an eye and a lens, respectively. Lower panels indicate higher magnification of lens epithelium area. No EGFP expression was observed in either wild-type or *rw341* mutant lens epithelium (le).

Scale bars: 20 µm.

Development • Supplementary information

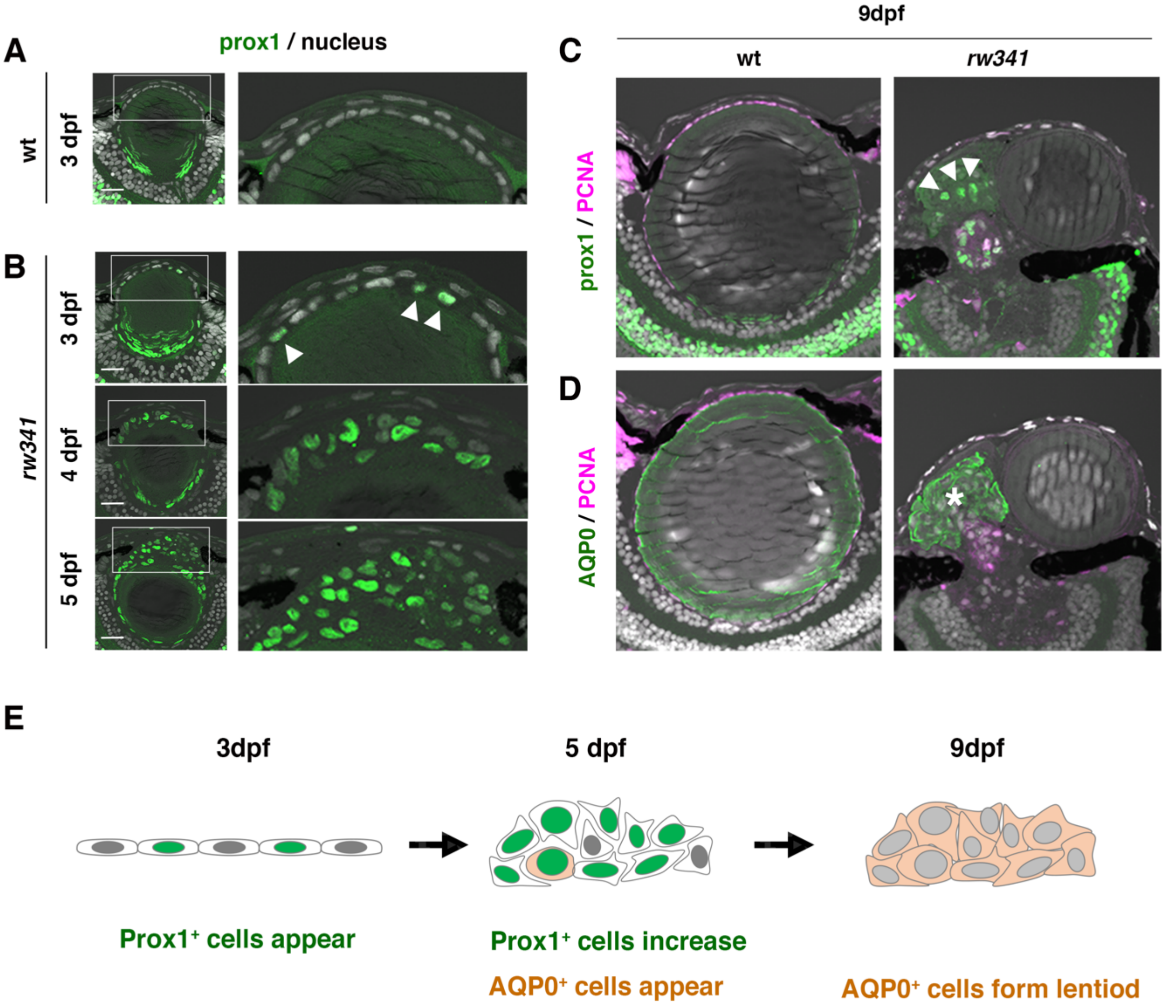

***Figure S2*: Lens epithelial cells enter fiber differentiation without passing through the equator in *rw341* mutants**

(A, B) Labeling of wild-type (A) and *rw341* mutant (B) lenses with anti-Prox1 antibody. Nuclei were counter-stained with TOPRO3. Right panels indicate higher magnification of lens epithelium area shown by squares in the left panels. In *rw341* mutants at 3dpf, some cells express Prox1 in monlayered lens epithelium (B, arrowheads) . Prox1-positive cells increase and the monolayer of lens epithelium is disrupted at 4 dpf.

(C, D) Prox1 (C) and AQP0 (D) expression in 9 dpf wild-type and *rw341* mutant lenses counter-labeled with anti-PCNA antibody. In *rw341* mutant lenses, aggregated anterior lens cells are observed close to the transparent lens fiber core. Only a few cells express Prox1 (C arrowheads), and most cells express AQP0 (D, asterisk). AQP0-positive cells are still nucleated, suggesting that denucleation process may be compromised.

(E) Developmental profile of lens phenotypes in *rw341* mutants. *rw341* mutant lens epithelial cells start to express Prox1 at 3 dpf, and then Prox1 expression switches to AQP0 expression after 5 dpf. Most cells express AQP0 to form lentoid at 9 dpf.

Scale: 20 µm (A, B).

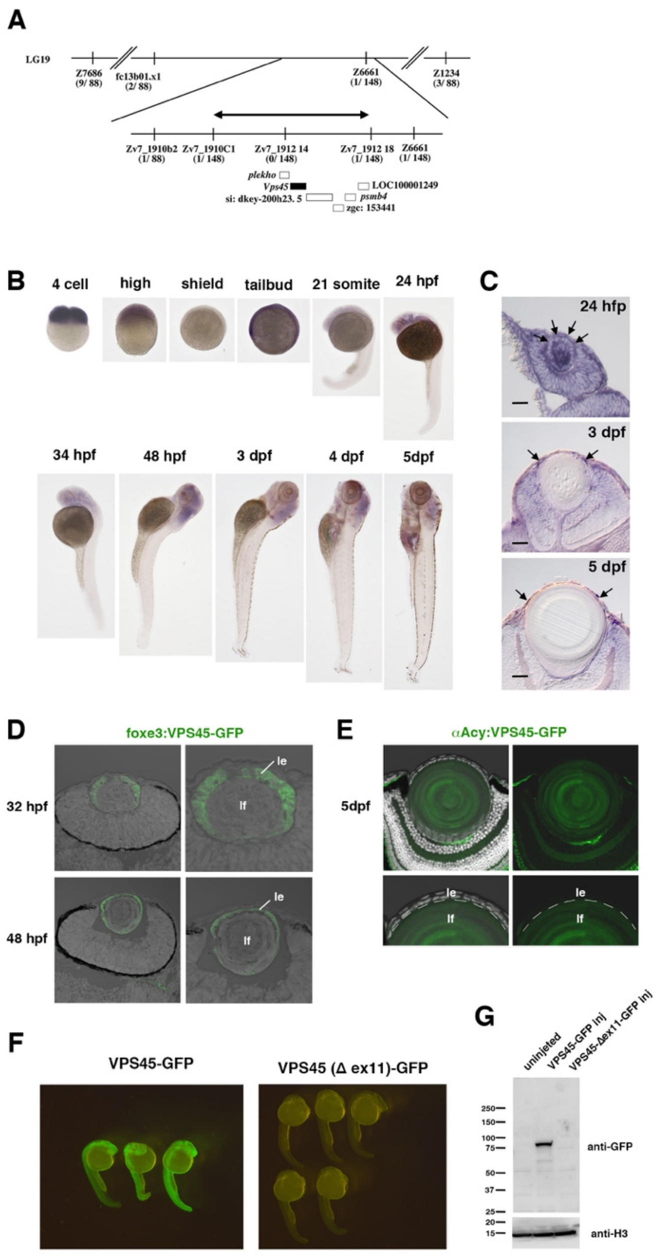

Figure S3: **Cloning of *rw341* mutant gene**

(A) The *rw341* mutation is mapped on chromosome 19 and is restricted within the genomic region, flanked by two polymorphic markers, Zv7_1912 14 and Zv7_1912 18 (arrow). In this region, six genes including *vps45* are annotated. Information regarding polymorphic markers is provided in Table S1.

(B) Whole mount *in situ* hybridization of wild-type embryos with the *vps45* mRNA probe during development.

(C) Section of wild-type lenses labeled with the *vps45* mRNA probe at 24hpf, 3 dpf and 5 dpf. Arrowheads indicate mRNA signals in lens vesicle (24 hpf) and lens epithelium (3 and 5 dpf).

(D) GFP expression in lenses of *Tg[foxe3:VPS45-GFP]* at 32 and 48 hpf. Right panels indicate higher magnification of lenses. GFP is expressed only in lens epithelium (le) but not in lens fiber core (lf).

(E) GFP expression in lenses of *Tg[αAcy:VPS45-GFP]* at 5 dpf. Bottom panels indicate higher magnification of the interface between lens epithelium and lens fiber core. GFP is expressed only in lens fiber core (lf) but not in lens epithelium (le).

(F) Embryos injected with mRNA encoding VPS45-GFP and VPS45(Δex11)-GFP. VPS45-GFP expression is maintained, but VPS45(Δex11)-GFP expression disappears at 24 hpf.

(G) Western blotting of 24 hpf wild-type embryos, and wild-type embryos injected with *vps45-GFP* mRNA and *vps45(Δex11)-GFP* mRNA with anti-GFP antibody. A band of GFP antibody is detected in *vps45-GFP* mRNA injected embryos, but disappears in *vps45(Δex11)-GFP* mRNA injected embryos.

Development • Supplementary information

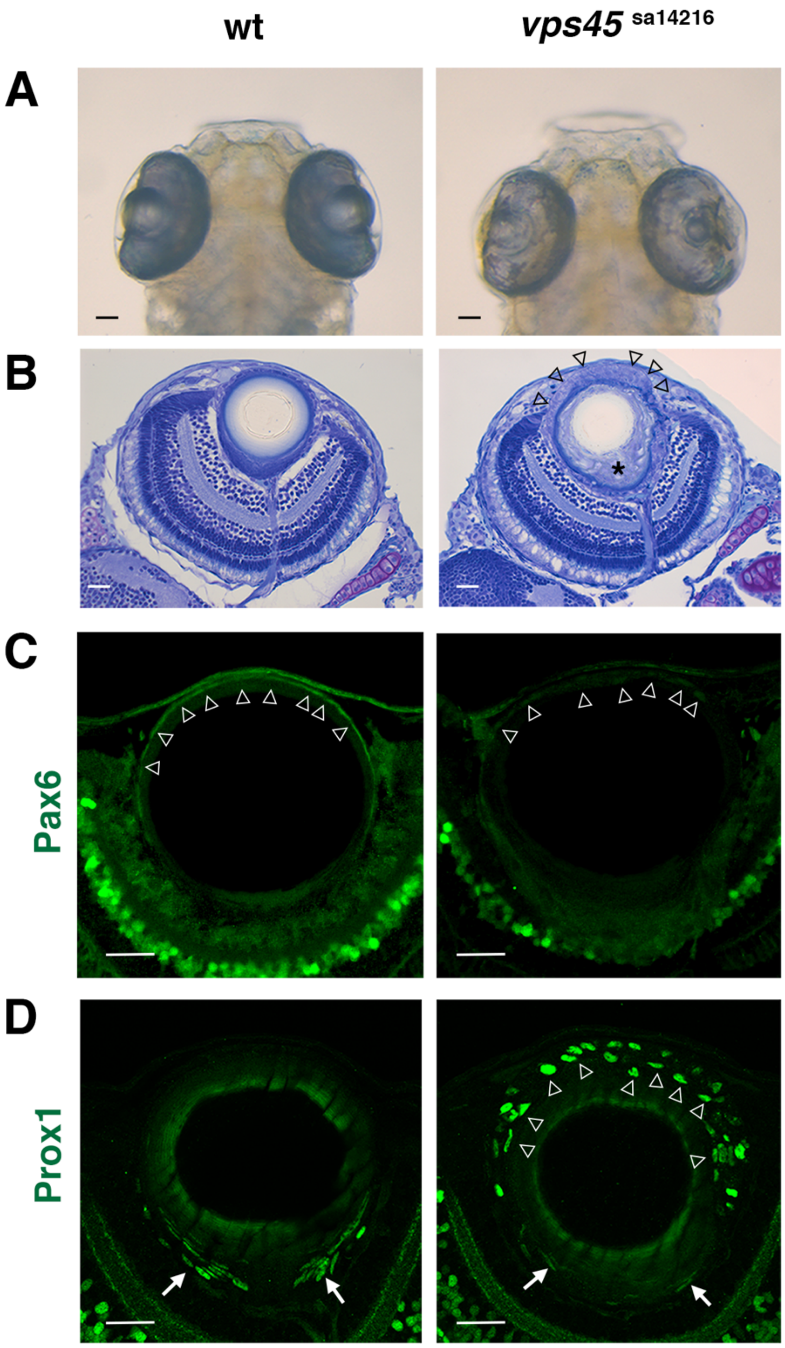

Figure S4: **Lens phenotypes of the zebrafish *vps45* non-sense mutant allele**

(A) Heads of wild-type and *vps45*^{sa14216} mutants. As in *rw341* mutants, a small lens fiber core is observed in the eye cup of *vps45*^{sa14216} mutants.

(B) Sections of wild-type and *vps45*^{sa14216} mutant eyes. Like *rw341* mutants, lens epithelium is disrupted to form multiple layers in *vps45*^{sa14216} mutants (arrowheads). Suture formation of elongating lens fibers is also abnormal in *vps45*^{sa14216} mutants (asterisk).

(C) Pax6 expression (green) in wild-type and *vps45*^{sa14216} mutant lenses. In wild type, Pax6 is expressed in a monolayer of lens epithelial cells (arrowheads). In *vps45*^{sa14216} mutants, Pax6 expression was detected, but becomes weaker than in wild type lenses (arrowheads).

(D) Prox1 expression (green) in wild-type and *vps45*^{sa14216} mutant lenses. In wild type lenses, Prox1 is expressed only in early differentiating lens fiber cells (arrows). In *vps45*^{sa14216} mutants, Prox1 is ectopically expressed in multilayered anterior lens epithelium (arrowheads).

Scale bars: 20 μm.

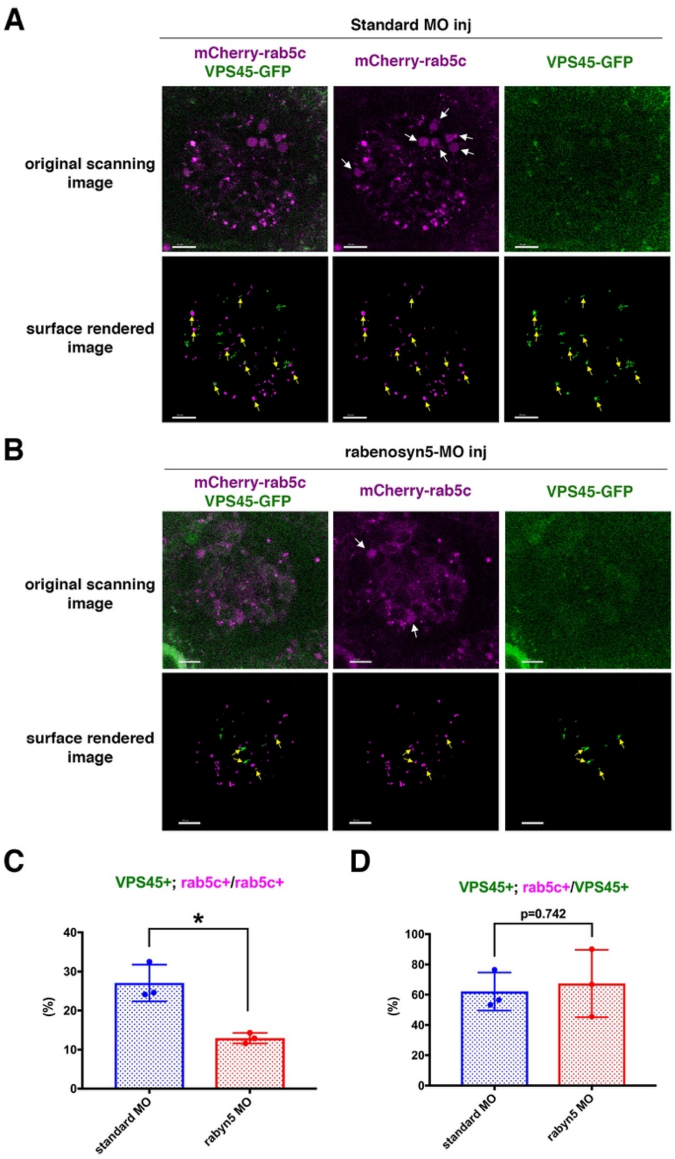

Figure S5: **VPS45 is localized to rab5-positive early endosomes due to the action of rabenosyn-5**

(a) Confocal scanning of lens epithelium of wild-type embryos injected with standard-MO and a mixture of *vps45-GFP* (green) and *mCherry-rab5c* (magenta) mRNA. Strong dotted mCherry-rab5c signals are observed. Weak VPS45-GFP signals are ubiquitously observed, but strong dotted peaks are also detected. White arrows indicate dying cells, which emitted auto-fluorescence overlapped with mCherry signals. Bottom panels indicate surface rendering images prepared with Imaris software, in which auto-fluorescent signals linked to dying cells were manually eliminated. Green surface-rendering objects were extracted from only dotted signals of VPS45-GFP by eliminating weak background signals. Yellow arrows indicate VPS45-GFP/mCherry-rab5c double-positive signals.

(b) Confocal scanning of lens epithelium of *rabenosyn-5* morphant embryos injected with a mixture of *vps45-GFP* (green) and *mCherry-rab5c* (magenta) mRNA. Like standard-MO injected embryos, strong dotted signals of mCherry-rab5c were observed. White arrows indicate auto-fluorescence from dying cells. However, there were almost no strong dotted signals of VPS45-GFP. Surface rendering images prepared with Imaris software are shown at the bottom. Auto-fluorescent signals linked to dying cells were manually eliminated. Yellow arrows indicate VPS45-GFP/mCherry-rab5c double-positive signals, the number of which was much lower than that of standard-MO injected lenses.

(c) Percentage of VPS45-GFP and mCherry-rab5c double-positive foci relative to the number of mCherry-rab5c positive foci. Averages and standard deviations are indicated. The average percentage was 27.1% in standard-MO injected lens epithelium, whereas it was reduced to 12.9% in *rabenosyn-5* morphant lens epithelium.

(d) Percentages of VPS45-GFP and mCherry-rab5c double-positive foci relative to the number of VPS45-GFP positive foci. Averages and standard deviations are indicated. There is no statistical difference between standard-MO and *rabenosyn-5*-MO injected lens epithelium. This result is consistent the model that VPS45 is recruited and co-localize with rab5c-positive endosomes through the interaction of Rabenosyn-5.

Probability is calculated with Students' t-test (two sided, unpaired, Welch): *p<0.05.
Scale bars: 20 μm.

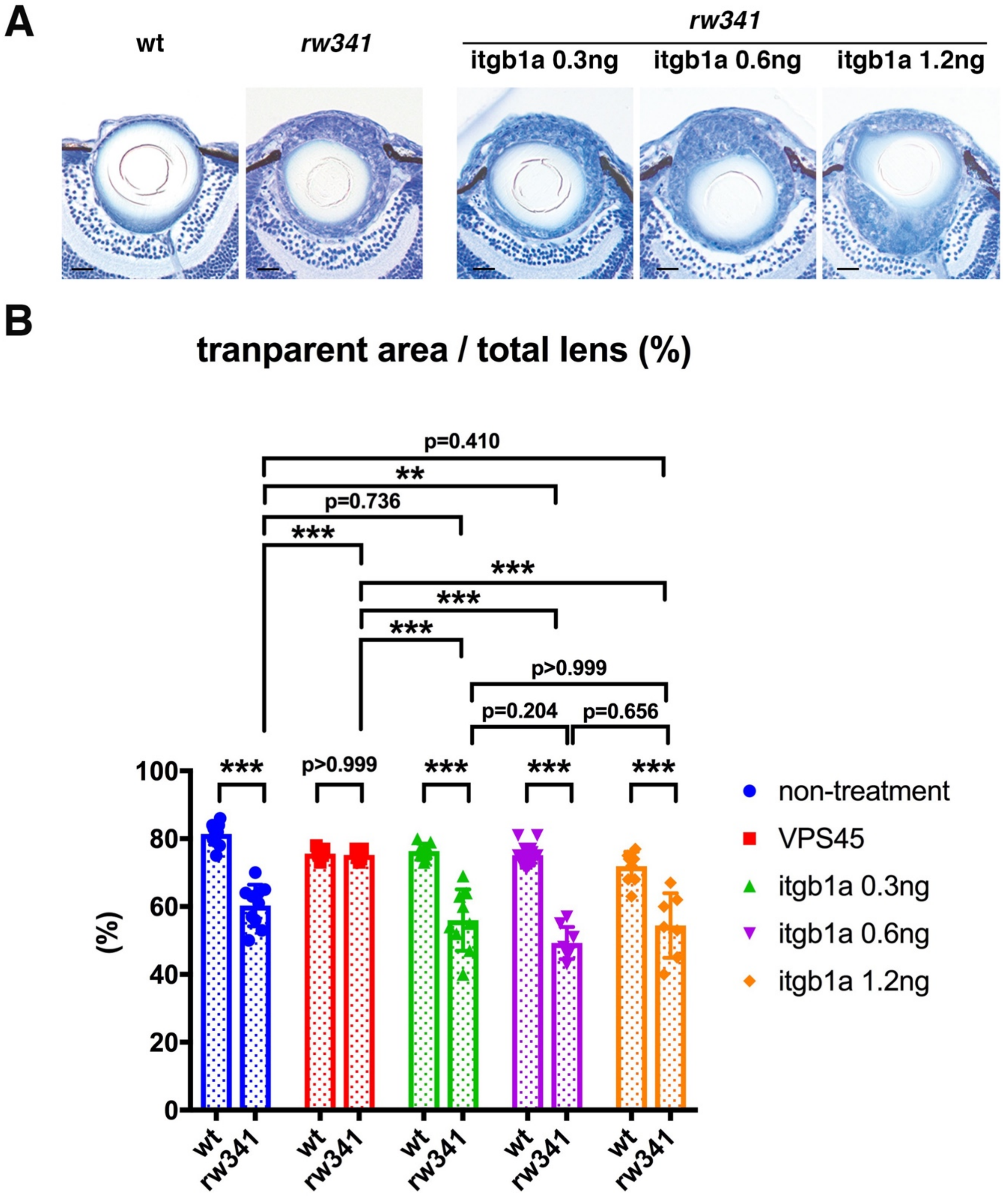

Figure S6: **Multilayered lens epithelial phenotypes are not rescued by overexpression of *integrin β1a* mRNA**

(A) Lens phenotypes of *rw341* mutant embryos injected with *integrin β1a* mRNA at 5 dpf. Multi-layered phenotypes of *rw341* mutants were not rescued by overexpression of *integrin β1a* mRNA. Scale bars: 20 μm.

(B) Percentage of transparent lens fiber area relative to the total lens area in wild-type, *rw341* mutants, *rw341* mutants injected with *vps45* mRNA, and *rw341* mutants injected with *integrin β1a* mRNA. Averages and standard deviations are indicated. Overexpression of *vps45* mRNA significantly recovered the transparent area in *rw341* mutants. However, overexpression of *integrin β1a* mRNA did not. Two-way ANOVA, multiple comparison using Turkey: **p<0.01, ***p<0.005.

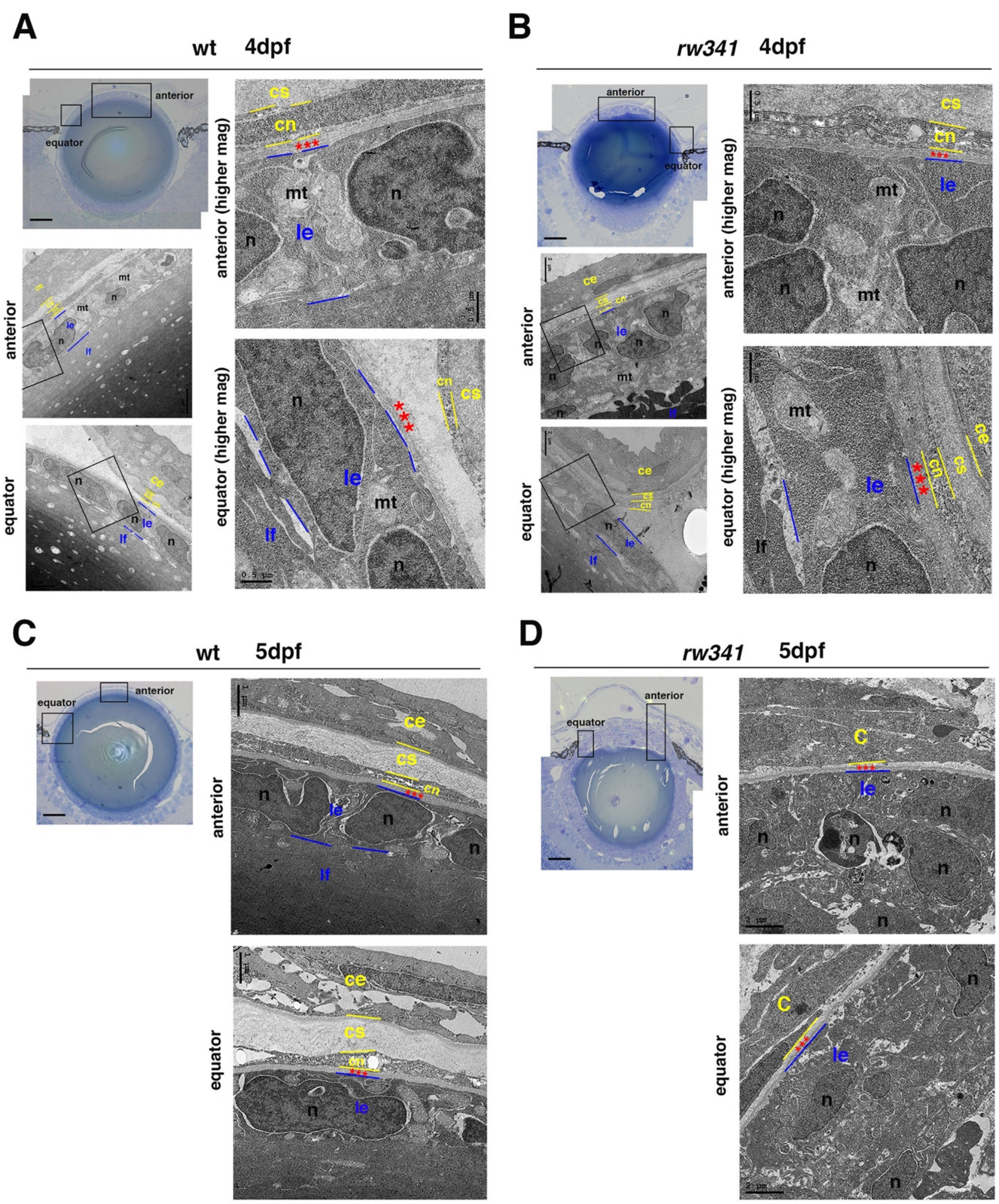

Figure S7: **Extracellular matrix seems to be normal in *rw341* mutant lens epithelium**

(A-D) Electron microscopic analyses of wild-type and *rw341* mutant lens at 4 dpf and 5 dpf. Anterior and equatorial regions of lens epithelium are shown. Cornea epithelium (ce), cornea stroma (cs) and cornea endothelium (cn) are indicated in yellow. There is a layer between cornea endothelium and lens epithelium that corresponds to extracellular matrix tissue (red asterisks). In *rw341* mutants, this layer is maintained at both 4 and 5 dpf, although three cornea layers are not clearly distinct (c) at 5 dpf. c, cornea; le, lens epithelium; lf, lens fiber cells; n, nucleus; mt, mitochondria.

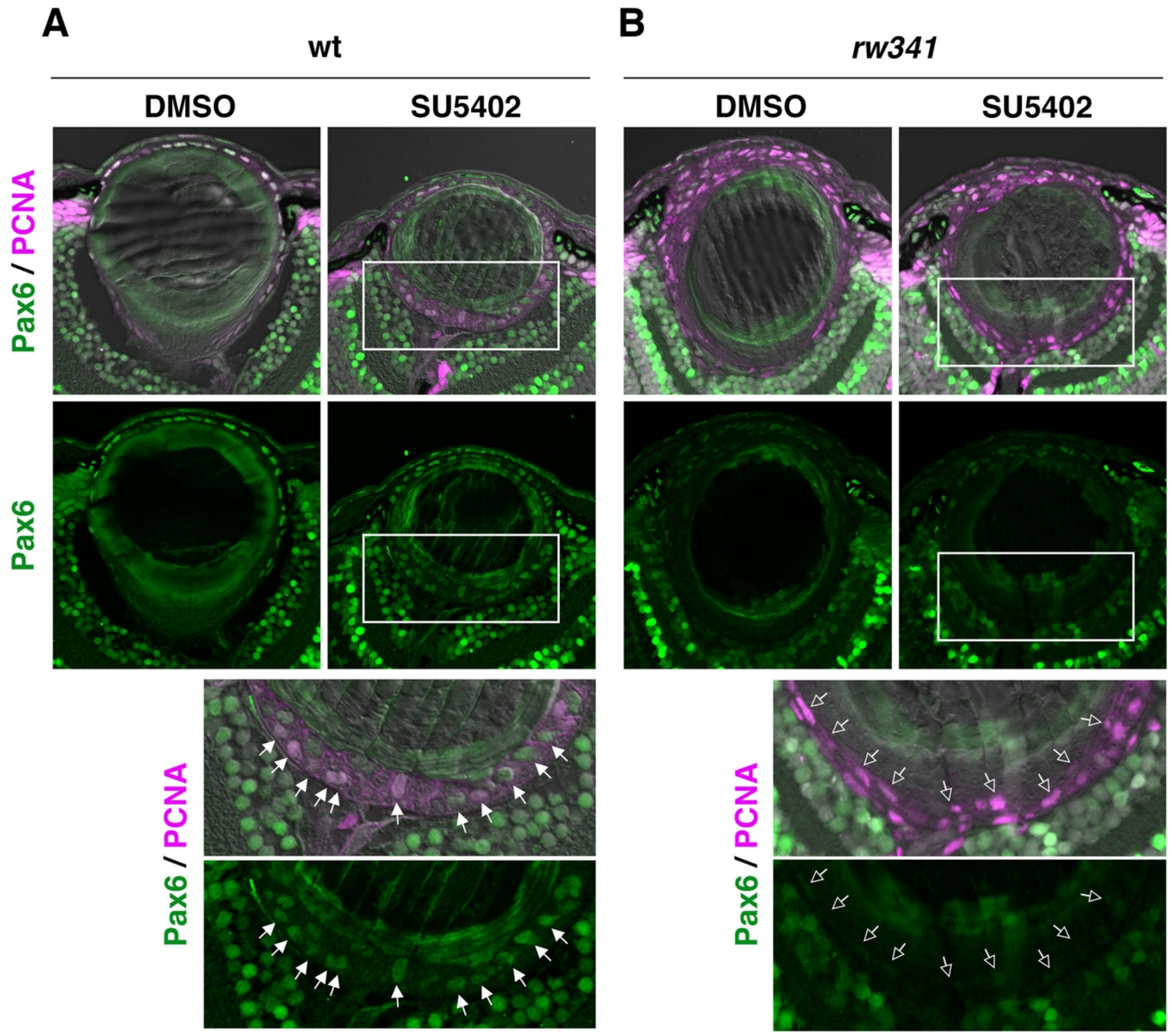

Figure S8: **Pax6 expression in SU5402-treated wild-type and *rw341* mutant lenses**

(A, B) Pax6 and PCNA expression of wild-type (A) and *rw341* mutant (B) lenses treated with either DMSO or SU5402. Bottom two large panels indicate higher magnification indicated in squares of the upper panels of SU5402-treated lenses. In DMSO-treated wild type, Pax6 is expressed in lens epithelium, and PCNA is expressed in lens epithelial cells as well as early differentiating lens fiber cells, the latter of which partially overlap with Prox1 expressing cells. In SU5402-treated wild-type lenses, many PCNA-positive cells are located along the posterior margin of the lens fiber region and these cells express Pax6 (A, arrows), suggesting that these PCNA-positive cells maintain lens epithelial cell fate. In DMSO- and SU5402-treated *rw341* mutants, Pax6 expression is low in anterior multilayered lens cells. SU5402-treated *rw341* mutants, many PCNA-positive cells accumulated along the margin of posterior lens fiber core, but, in contrast to SU5402-treated wild-type lenses, these cells did not express Pax6 (B, open arrows). Since these PCNA cells express Prox1 (Fig. 4C), they enter early stage of lens fiber cell differentiation.

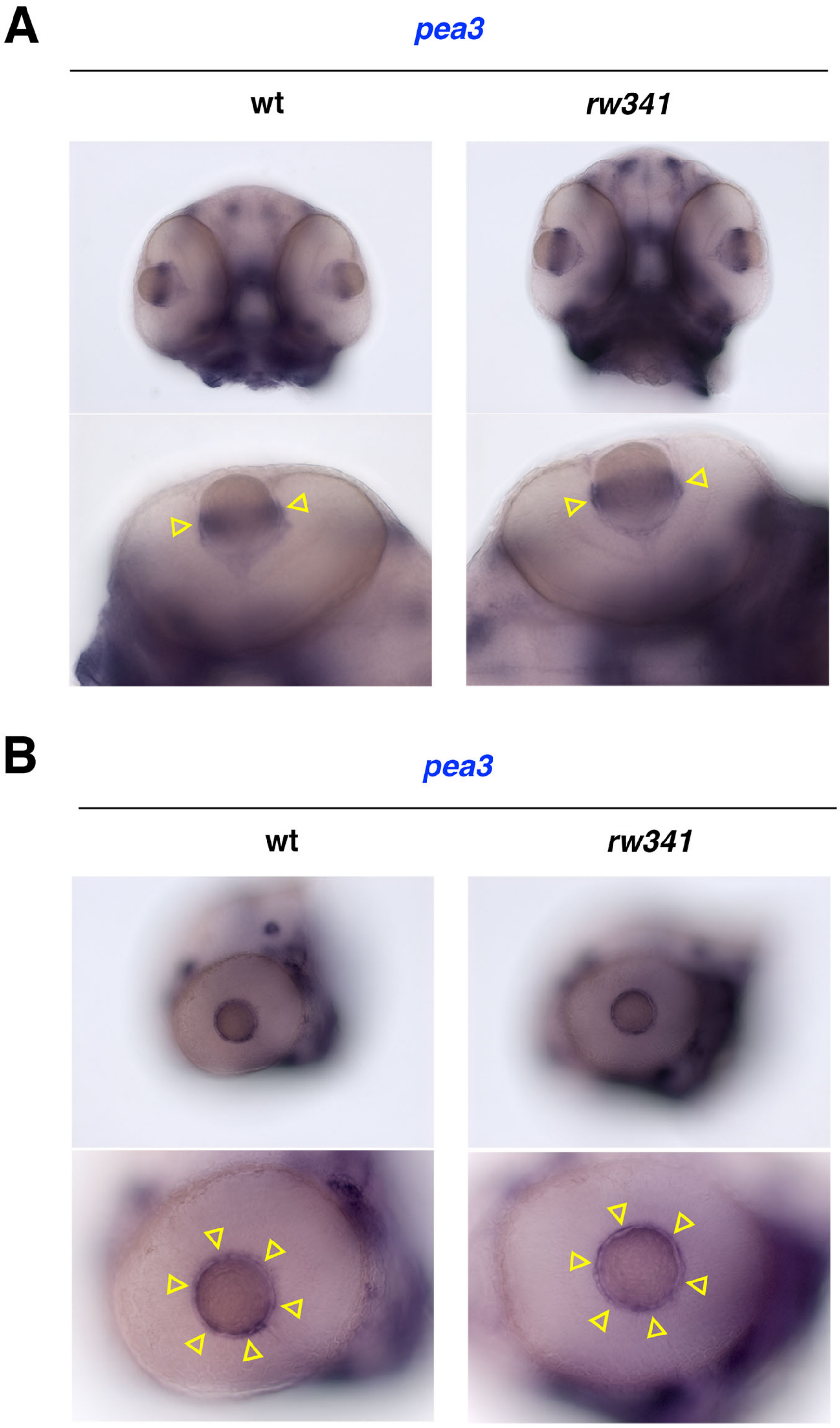

Figure S9: **FGF signaling is not altered in *rw341* mutant lenses**
(A, B) Ventral (A) and lateral (B) views of wild-type and *rw341* mutant heads labeled with *pea3* RNA probe. Lower panels show the eye. *pea3* mRNA is expressed along the circumference of the lens equator in wild type (yellow arrowheads). The spatial expression pattern of *pea3* mRNA is not changed in *rw341* mutants (yellow arrowheads).

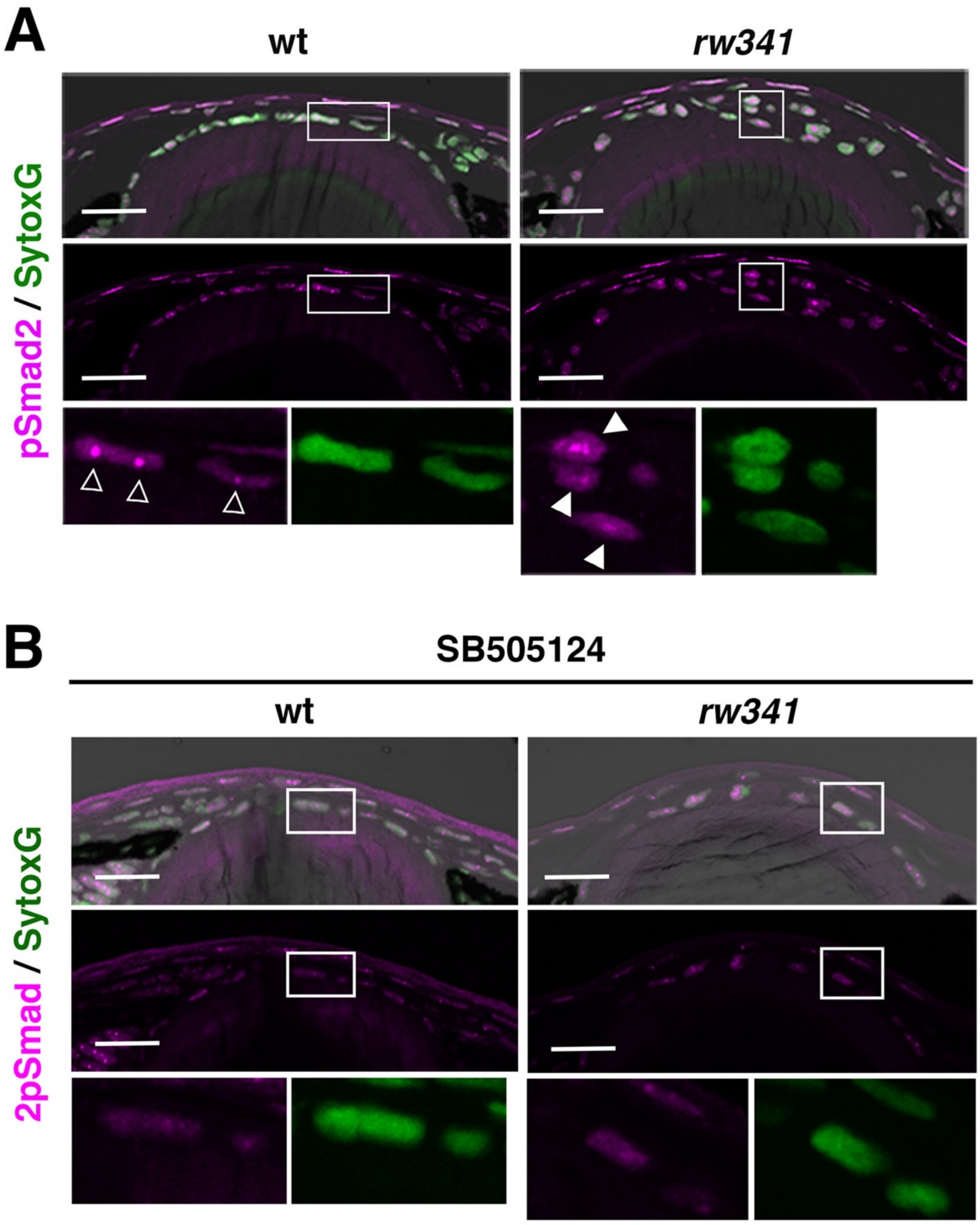

Figure S10: **SB505124 treatment inhibits TGF-β signaling in zebrafish lens epithelium**

(A) Labeling of 5 dpf wild-type and *rw341* mutant lenses with anti-pSmad2 antibody (magenta) and Sytox Green (green). Upper panels show lens epithelium. Middle panels indicate the magenta channel. Bottom panels show higher magnifications of squares indicated in the upper/middle panels. In wild-type lenses, small dotted pSmad2 signals are observed in lens epithelial cell nuclei (open arrowheads). In *rw341* mutants, pSmad2 signals increased throughout the whole region of lens epithelial cell nuclei (filled arrowheads).

(B) Labeling of 5 dpf SB505124-treated wild-type and *rw341* mutant lenses with anti-pSmad2 antibody (magenta) and Sytox Green (green). Upper panels show lens epithelium. Middle panels indicate the magenta channel. Bottom panels show higher magnification of squares indicated in the upper/middle panels. SB505124 treatment markedly reduces the intensity of pSmad2 signals in both wild type and *rw341* mutant lens epithelium, confirming that this concentration of SB505124 effectively inhibits TGF-β signaling in lens epithelium.

Scale bars: 20 μm (A, B).

Development • Supplementary information

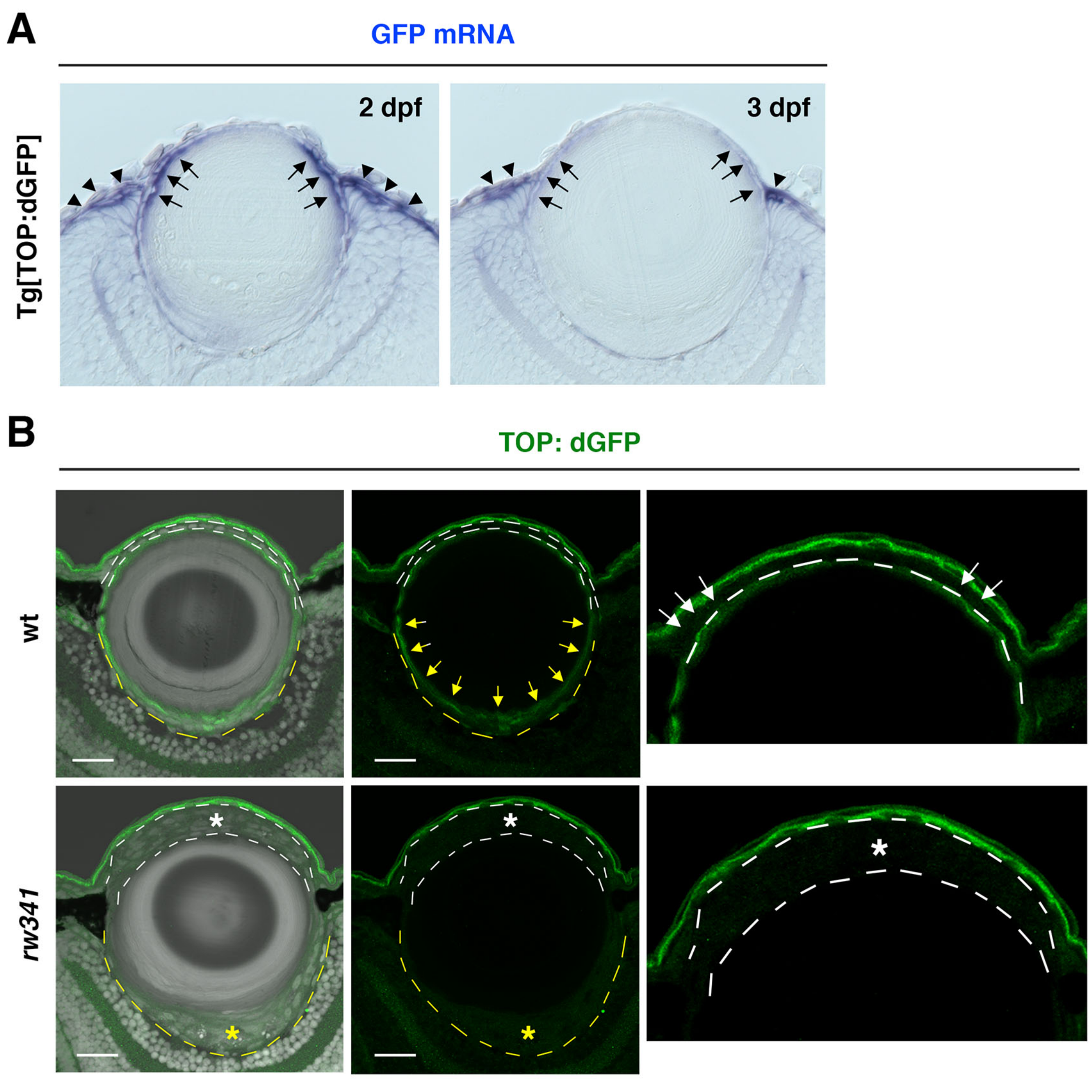

Figure S11: **Canonical Wnt signaling activity in lens epithelium is inhibited in *rw341* mutants**

(A) dGFP mRNA expression (blue) in Tg[TOP:dGFP] transgenic wild-type lenses at 2 and 3 dpf. dGFP mRNA is expressed in the peripheral region of lens epithelium (arrows) as well as pigment epithelium associated with retinal CMZ (arrowheads).

(B) dGFP protein expression (green) of wild-type and *rw341* mutant lenses carrying the transgene Tg[TOP:dGFP] at 5 dpf. Nuclei were counter-stained with TOPRO (white). Middle panels indicate only the green channel. Right panels show higher magnification of the anterior lens area. White dotted lines indicate lens epithelium in wild-type and multilayered anterior lens cells in *rw341* mutants. The yellow dotted line indicates the peripheral edge of the posterior lens fiber core. Yellow arrows indicate dGFP signals in early differentiating lens fiber cells in wild-type lenses (middle panels). Very faint dGFP signals were detected in peripheral lens epithelium corresponding to the germinative zone in wild-type lenses (white arrows in the right panels), where dGFP mRNA is expressed (A). There is no dGFP signal in anterior multilayered lens cells (white asterisk) and posterior lens fiber area (yellow asterisk) in *rw341* mutants. Scale bars: 20 µm.

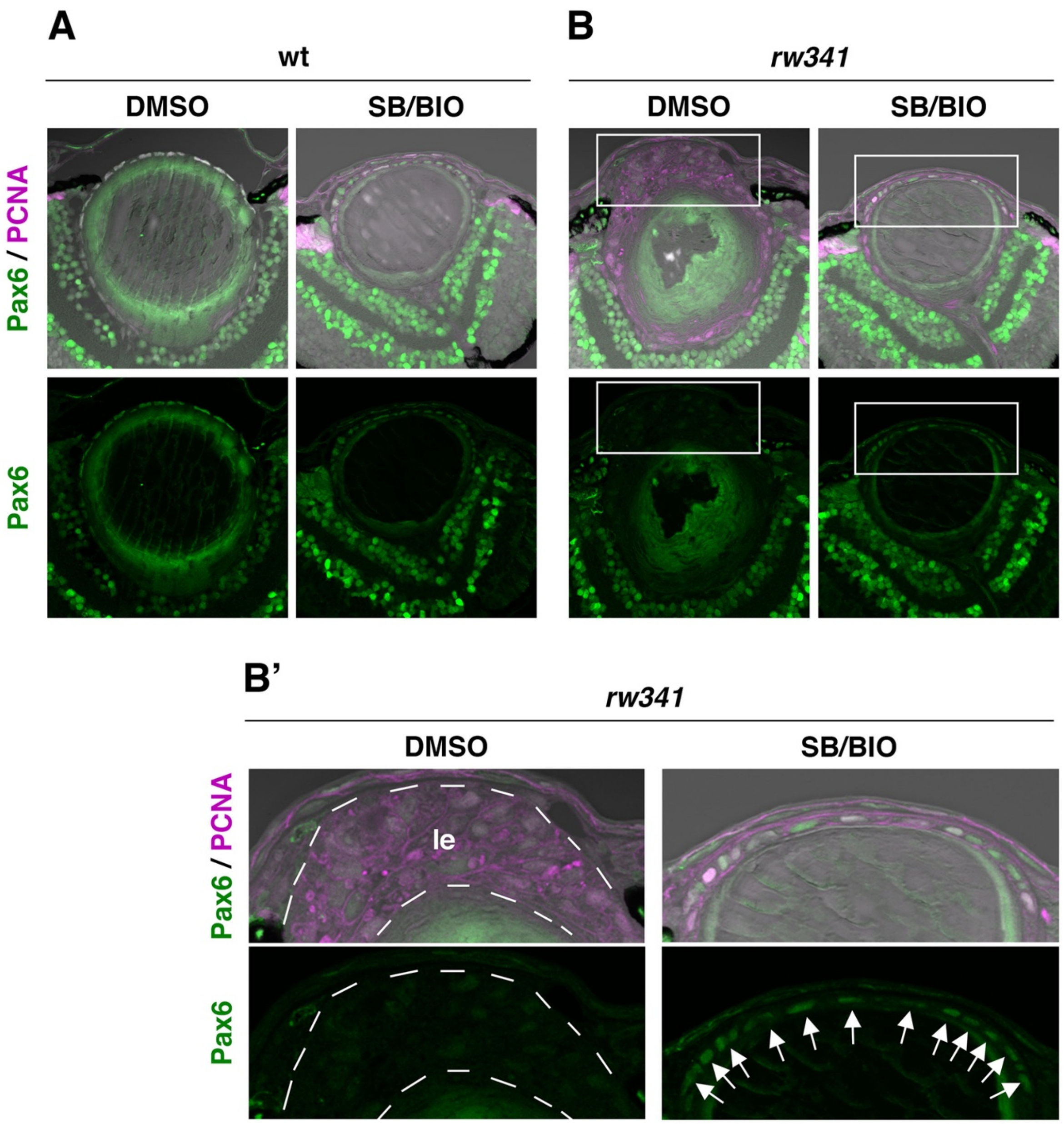

Figure S12: **Pax6 expression in SB505124 and BIO-treated wild-type and *rw341* mutant lenses**
(A, B) Pax6 and PCNA expression of wild-type (A) and *rw341* mutant (B) lenses. Panels in (B') indicate higher magnification images of squares in (B), which cover lens epithelium. In DMSO-treated wild type, Pax6 is expressed in lens epithelium. SB505124/BIO-treatment did not affect Pax6 expression in wild-type lenses. In DMSO-treated *rw341* mutants, Pax6 expression is low in anterior multilayered lens cells (B', le). SB505124/BIO-treated *rw341* mutants, lens epithelium is monolayered and express Pax6 (B', arrows).

Table S1: **New polymorphic markers linked to *rw341* mutation**

Name	Forward primer	Reverse primer	Position of LG19 (Zv_7)
Zv7_1910b2	CATAGTGGTGATGTTGATGTGC	GCTGCTTCATTGATGTGCTC	38773917
Zv7_1910C1	CATTGTGGGACACATACTTCTG	TTGCTGAGCTGGACTGAAGA	38986809
Zv7_1912 14	GCACCAGGTCAAACACTTCA	GCTTCAGGCCTGGTGATG	39184593
Zv7_1912 18	AAATACTTTAGGGAAAACATCAAAAA	GCATCCTCTGAAGCCATGAT	39304799

Development • Supplementary information

Table S2. Gene annotation in the zebrafish genome database

gene name (ZFIN)	Ensemble (GRCz11)		Genbank accession
vps45	vsp45	ENSDARG00000061180	NM_001256656
rabenosyn-5	rbsn	ENSDARG00000078215	CR929302
rab5aa	rab5aa	ENSDARG00000018602	NM_201485
rab5c	rab5c	ENSDARG00000026712	NM_201501
rab7	rab7	ENSDARG00000020497	NM_200928
rab11a	rab11a	ENSDARG00000041450	NM_001007359
integrin β1a	itgb1a	ENSDARG00000071863	NM_001034971
pea3	etv4	ENSDARG00000018303	NM_131425
αSMA	acta2	ENSDARG00000045180	NM_212620
foxe3	foxe3	ENSDARG00000062892	NM_001079682
αA crystallin	cryaa	ENSDARG00000053502	NM_152950

Development • Supplementary information

Table S3. Sample size and *P*-value

p<0.05	student t-test, unpaired, Welch correction
p>0.05	One way ANOVA, Turkey
	Two way ANOVA Turkey

Fig. 1J

	n	wt	rw341
wt	9		
rw341	11	0.3473	

Fig. 2C

	n	wt	rw341
wt	5		
rw341	3	0.0004	
wt+VPS45 0.5ng	6		
rw341+VPS45 0.5ng	3	0.9953	0.0039
wt+VPS45-Δex11 0.5ng	5		
rw341+VPS45-Δex11 0.5ng	3	<0.0001	0.1107

Fig. 2E

	n	wt	rw341
wt	4		
rw341	4	<0.0001	
rw341; Tg(foxe3:VPS45-GFP)	4	0.5375	0.0001

Fig. 2G

	n	wt	rw341
wt	4		
rw341	15	<0.0001	
rw341; Tg(αcry:VPS45-GFP)	6	0.0003	0.0113

Fig. 3B

	n	wt	rw341	rw341+VPS45 0.3ng
wt	5			
rw341	5	0.0001		
wt+VPS45 0.3ng	4			
rw341+VPS45 0.3ng	4	0.3932	0.0174	
wt+rabenosyn5 0.3ng	3			
rw341+rabenosyn5 0.3ng	4	0.1577	0.0580	0.9929

Fig. 3D

	n	wt	rw341	rw341+rab5aa 0.4ng	rw341+rab5c 0.4ng
wt	6				
rw341	6	<0.0001			
wt+rab5aa 0.4 ng	6				
rw341+rab5aa 0.4ng	5	0.0006	0.1271		
wt+rab5c 0.4 ng	7				
rw341+rab5c 0.4ng	4	0.0375	0.0114	0.9474	
wt+rab11a 0.4 ng	4				
rw341+rab11a 0.4 ng	7	0.0027	0.0073	0.9875	>0.9999

Fig. 3G

	n of cells	n of embryos	wt
wt	6	4	
rw341	12	4	0.0921

Fig. 3H

	n of cells	n of embryos	wt
wt	8	7	
rw341	8	4	0.0328

Fig. 3I

	n of cells	n of embryos	wt
wt	8	4	
rw341	10	5	<0.0001

Fig. 4E

	n	wt+DMSO	rw341+DMSO	wt+SU5402
wt+DMSO	4			
rw341+DMSO	4	0.0061		
wt+SU5402	4	>0.9999		
rw341+SU5402	4		0.9966	0.0042

Fig. 4F

	n	rw341+DMSO
rw341+DMSO	4	
rw341+SU5402	4	0.8624

Fig. 4G

	n	wt+DMSO	rw341+DMSO	wt+SU5402
wt+DMSO	4			
rw341+DMSO	4	0.2494		
wt+SU5402	4	0.0007		
rw341+SU5402	4		0.9948	0.0125

Fig. 5B

	n	wt	rw341	wt+DMSO	rw341+DMSO	wt+SB505124
wt	14					
rw341	18	<0.0001				
wt+DMSO	18	0.9961				
rw341+DMSO	18		0.0157	<0.0001		
wt+SB505124	18	0.9878		>0.9999		
rw341+SB505124	36		<0.0001		<0.0001	<0.0001

Fig. 5E

	n	rw341+DMSO
rw341+DMSO	6	
rw341+SB505124	6	0.0060

Fig. 5F

	n	rw341+DMSO
rw341+DMSO	6	
rw341+SB505124	6	0.0001

Fig. 6B

	n	rw341+DMSO	rw341+BIO	rw341+SB505124
rw341+DMSO	6			
rw341+BIO	6	0.0100		
rw341+SB505124	4	0.0012	0.6350	
rw341+BIO+SB505124	16	<0.0001	0.0022	0.1942

Fig. 6C

	n	rw341+DMSO	rw341+BIO	rw341+SB505124
rw341+DMSO	6			
rw341+BIO	6	0.0204		
rw341+SB505124	4	0.2408	0.8221	
rw341+BIO+SB505124	16	<0.0001	0.0010	0.0004

Fig. S5C

	n	standard MO
standard MO	3	
rabyn5 MO	3	0.0275

Fig. S5D

	n	standard MO
standard MO	3	
rabyn5 MO	3	0.7422

Fig. S6B

	n	wt	rw341	wt+VPS45	rw341+VPS45	wt+itb1a 0.3g	rw341+itb1a 0.3ng	wt+itb1a 0.6g	rw341+itb1a 0.6ng	wt+itb1a 1.2g
wt	10									
rw341	10	<0.0001								
wt+VPS45	5									
rw341+VPS45	7		<0.0001	>0.9999						
wt+itb1a 0.3g	9									
rw341+itb1a 0.3ng	9		0.7361		<0.0001	<0.0001				
wt+itb1a 0.6g	14									
rw341+itb1a 0.6ng	8		0.0010		<0.0001		0.2064	<0.0001		
wt+itb1a 1.2g	10									
rw341+itb1a 1.2ng	7		0.4099		<0.0001		0.9998		0.6561	<0.0001

Table S4. Post-hoc achieved power (Student's t-test, two-tailed)

power>0.8
power<0.8

Fig. 1J

	n	wt	rw341
wt	9		
rw341	11		

Fig. 2C

	n	wt	rw341
wt	5		
rw341	3	1.0000000	
wt+VPS45 0.5ng	6		
rw341+VPS45 0.5ng	3		0.9997358
wt+VPS45-Δex11 0.5ng	5		
rw341+VPS45-Δex11 0.5ng	3	0.9861652	

Fig. 2E

	n	wt	rw341
wt	4		
rw341	4	1.0000000	
rw341; Tg(foxe3:VPS45-GFP)	4		0.9999842

Fig. 2G

	n	wt	rw341
wt	4		
rw341	15	0.9999999	
rw341; Tg(αcry:VPS45-GFP)	6	1.0000000	0.6646760

Fig. 3B

	n	wt	rw341	rw341+VPS45 0.3ng
wt	5			
rw341	5	0.9879854		
wt+VPS45 0.3ng	4			
rw341+VPS45 0.3ng	4		0.6431451	
wt+rabenosyn5 0.3ng	3			
rw341+rabenosyn5 0.3ng	4			

Fig. 3D

	n	wt	rw341	rw341+rab5aa 0.4ng	rw341+rab5c 0.4ng
wt	6				
rw341	6	0.9993187			
wt+rab5aa 0.4 ng	6				
rw341+rab5aa 0.4ng	5	0.9997833			
wt+rab5c 0.4 ng	7				
rw341+rab5c 0.4ng	4	0.9994263	0.6733749		
wt+rab11a 0.4 ng	4				
rw341+rab11a 0.4 ng	7	0.9310401	0.5651884		

Fig. 3G

	n of cells	n of embryos	wt
wt	6	4	
rw341	12	4	

Fig. 3H

	n of cells	n of embryos	wt
wt	8	7	
rw341	8	4	0.5755303

Fig. 3I

	n of cells	n of embryos	wt
wt	8	4	
rw341	10	5	1.0000000

Fig. 4E

	n	wt+DMSO	rw341+DMSO	wt+SU5402
wt+DMSO	4			
rw341+DMSO	4	0.9010570		
wt+SU5402	4			
rw341+SU5402	4			0.9482191

Fig. 4F

	n	rw341+DMSO
rw341+DMSO	4	
rw341+SU5402	4	

Fig. 4G

	n	wt+DMSO	rw341+DMSO	wt+SU5402
wt+DMSO	4			
rw341+DMSO	4			
wt+SU5402	4	1.0000000		
rw341+SU5402	4			0.9535651

Fig. 5B

	n	wt	rw341	wt+DMSO	rw341+DMSO	wt+SB505124
wt	14					
rw341	18	1.0000000				
wt+DMSO	18					
rw341+DMSO	18		0.9721627	1.0000000		
wt+SB505124	18					
rw341+SB505124	36		1.0000000		0.9976035	1.0000000

Fig. 5E

	n	rw341+DMSO
rw341+DMSO	6	
rw341+SB505124	6	0.9568266

Fig. 5F

	n	rw341+DMSO
rw341+DMSO	6	
rw341+SB505124	6	0.9999966

Fig. 6B

	n	rw341+DMSO	rw341+BIO	rw341+SB505124
rw341+DMSO	6			
rw341+BIO	6	0.9994572		
rw341+SB505124	4	0.9034521		
rw341+BIO+SB505124	16	0.9999960	0.9766542	0.9930536

Fig. 6C

	n	rw341+DMSO	rw341+BIO	rw341+SB505124
rw341+DMSO	6			
rw341+BIO	6	0.8356313		
rw341+SB505124	4			
rw341+BIO+SB505124	16	1.0000000	0.9311570	0.9967939

Fig. S5C

	n	standard MO
standard MO	3	
rabyn5 MO	3	0.9396777

Fig. S5D

	n	standard MO
standard MO	3	
rabyn5 MO	3	

Fig. S6B

	n	wt	rw341	wt+VPS45	rw341+VPS45	wt+itb1a 0.3g	rw341+itb1a 0.3ng	wt+itb1a 0.6g	rw341+itb1a 0.6ng	wt+itb1a 1.2g
wt	10									
rw341	10	1.0000000								
wt+VPS45	5									
rw341+VPS45	7		0.9997729							
wt+itb1a 0.3g	9									
rw341+itb1a 0.3ng	9				0.9948940	0.9999083				
wt+itb1a 0.6g	14									
rw341+itb1a 0.6ng	8		0.9667596		1.0000000			1.0000000		
wt+itb1a 1.2g	10									
rw341+itb1a 1.2ng	7				0.9915218					0.9905190
